# Glucocorticoid receptor-NECAB1 axis can negatively regulate insulin secretion in pancreatic β-cells

**DOI:** 10.1038/s41598-023-44324-y

**Published:** 2023-10-20

**Authors:** Haruhide Udagawa, Nobuaki Funahashi, Wataru Nishimura, Takashi Uebanso, Miho Kawaguchi, Riku Asahi, Shigeru Nakajima, Takao Nammo, Masaki Hiramoto, Kazuki Yasuda

**Affiliations:** 1 Department of Metabolic Disorder, Diabetes Research Center, Research Institute, National Center for Global Health and Medicine, Shinjuku-ku, Tokyo 162-8655 Japan; 2 Department of Registered Dietitians, Faculty of Health and Nutrition, Bunkyo University, 1100 Namegaya, Chigasaki, Kanagawa 253-8550 Japan; 3 Department of Life Science and Technology, Tokyo Institute of Technology, Yokohama, Japan; 4 Department of Molecular Biology, International University of Health and Welfare School of Medicine, Narita, Chiba 286-8686 Japan; 5 Division of Anatomy, Bio-Imaging and Neuro-Cell Science, Jichi Medical University, Shimotsuke, Tochigi 329-0498 Japan; 6 Department of Preventive Environment and Nutrition, Institute of Biomedical Sciences, Tokushima University Graduate School, Tokushima, 770-8503 Japan; 7 Department of Metabolic Medicine, Graduate School of Medicine, Osaka University, Suita, Japan; 8 Department of Diabetes Care Medicine, Graduate School of Medicine, Osaka University, Suita, Japan; 9 Department of Biochemistry, Tokyo Medical University, Tokyo, 160-8402 Japan; 10 Department of Diabetes, Endocrinology and Metabolism, Kyorin University School of Medicine, 6-20-2 Shinkawa, Mitaka, Tokyo 181-8611 Japan

**Keywords:** Endocrinology, Endocrine system and metabolic diseases

## Abstract

The mechanisms of impaired glucose-induced insulin secretion from the pancreatic β-cells in obesity have not yet been completely elucidated. Here, we aimed to assess the effects of adipocyte-derived factors on the functioning of pancreatic β-cells. We prepared a conditioned medium using 3T3-L1 cell culture supernatant collected at day eight (D8CM) and then exposed the rat pancreatic β-cell line, INS-1D. We found that D8CM suppressed insulin secretion in INS-1D cells due to reduced intracellular calcium levels. This was mediated by the induction of a negative regulator of insulin secretion—NECAB1. LC–MS/MS analysis results revealed that D8CM possessed steroid hormones (cortisol, corticosterone, and cortisone). INS-1D cell exposure to cortisol or corticosterone increased *Necab1* mRNA expression and significantly reduced insulin secretion. The increased expression of *Necab1* and reduced insulin secretion effects from exposure to these hormones were completely abolished by inhibition of the glucocorticoid receptor (GR). NECAB1 expression was also increased in the pancreatic islets of *db/db* mice. We demonstrated that the upregulation of NECAB1 was dependent on GR activation, and that binding of the GR to the upstream regions of *Necab1* was essential for this effect. NECAB1 may play a novel role in the adipoinsular axis and could be potentially involved in the pathophysiology of obesity-related diabetes mellitus.

## Introduction

Type 2 diabetes mellitus (T2D) is a multifactorial disorder caused by both genetic and environmental factors. While more than 100 susceptibility loci for T2D have been identified by genome-wide association studies (GWAS) to date^1^, research focused on environmental factors is also required to elucidate the pathogenesis of T2D. Several risk factors for T2D have been identified, including age, sex, obesity, visceral fat accumulation, low physical activity, and smoking; among them, obesity is the main factor responsible for the onset and progression of diabetes^2^.

The pathophysiology of T2D with obesity as the dominant risk factor is mainly characterized by insulin resistance in peripheral organs and the insufficient secretion of insulin from pancreatic β-cells^3^. Adipose tissue is an important endocrine organ that secretes many biologically active substances, such as leptin, adiponectin, tumor necrosis factor (TNF), and monocyte chemoattractant protein 1 (MCP-1)^4–6^. Adipose tissue dysfunction in the obesogenic condition could be a main contributor to insulin resistance^7^. It has been suggested that inflammatory changes due to the dysregulated production of pro-inflammatory and anti-inflammatory adipocytokines within the adipose tissue may also contribute to the development of diabetes^8–10^. Interestingly, increased 11β-HSD1 expression, which contributes to the production of steroid hormones, has been reported in the adipose tissue of obese individuals^11^. It has been suggested that obesity-induced increases in HSD11B in adipose tissue may increase cortisol secretion and may contribute to an increase in local glucocorticoid signaling^12^. In addition, in obesity-associated T2D, impaired insulin secretion is considered essential for the onset of diabetes. It is also possible that adipocyte-derived factors induced by obesity, such as glucocorticoids, directly affect pancreatic β-cell function, although the pathophysiology of this process is poorly understood. We hypothesized that adipocyte-derived factors may be involved not only in insulin resistance, but also in insulin secretory disorder.

The discovery of new molecules involved in direct pancreatic β-cell function by adipocyte-derived factors is likely to lead to the development of new therapeutic drugs. In this study, to investigate the influence of adipocyte-derived factors on the function of pancreatic β-cells, we prepared a conditioned medium (CM) from supernatants of cultured adipocytes, and exposed the pancreatic β-cell line, INS-1D, to CM. Using this model, we found that CM suppressed insulin secretion in INS-1D cells, and this effect was mediated by the induction of a novel negative regulator of insulin secretion, NECAB1, dependent on glucocorticoid receptor (GR) activation. We also suggest that the binding of the GR to upstream regions of *Necab1* was essential for this effect.

## Results

### Adipocyte-derived conditioned medium decreased insulin secretion in INS-1D cells

**Figure 1:**
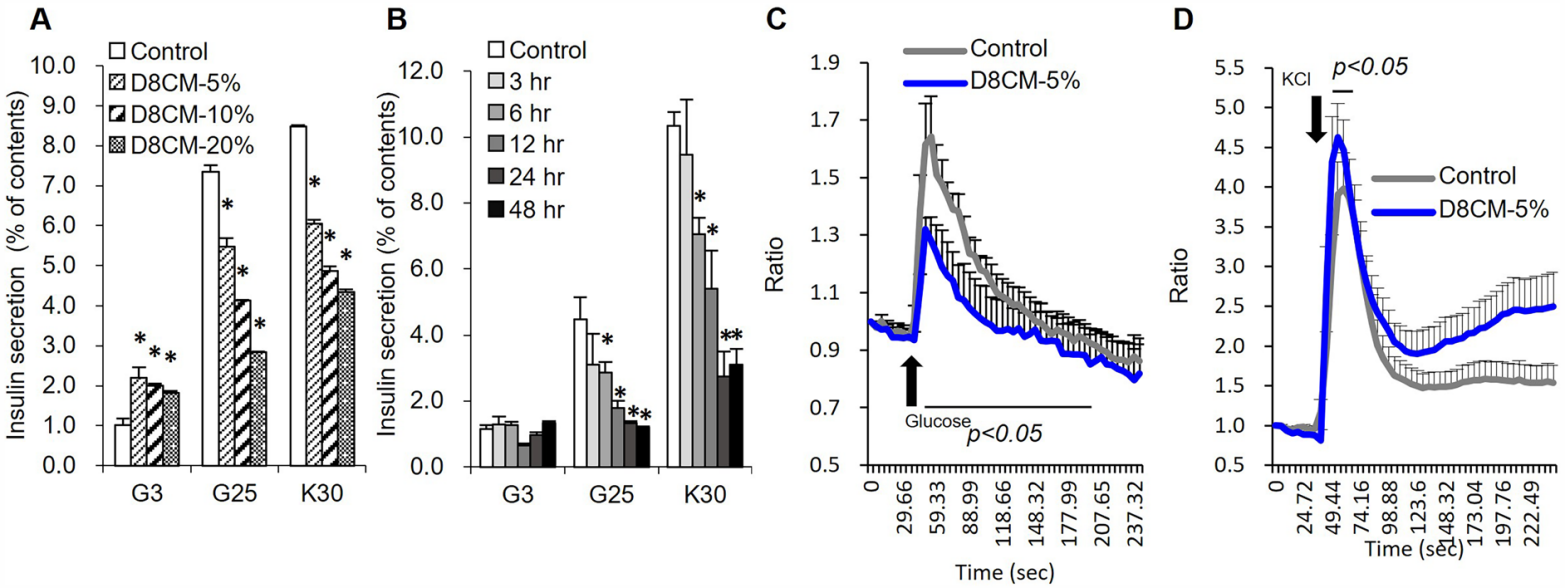
Impaired insulin secretion of INS-1D cells exposed to adipocyte-derived conditioned medium (D8CM). (**A**) Insulin secretion from INS-1D cells exposed to 5%, 10% and 20% day eight conditioned medium (D8CM) for 24 h was measured after treating with 3 mM glucose (G3), 25 mM glucose (G25) or 30 mM KCl (K30). (**B**) Insulin secretion from INS-1D cells exposed to 5% D8CM for 3, 6, 12, 24, 48 h were measured after treatment with G3 or G25 or K30. Insulin secretion is shown as percent of insulin content (secreted insulin/(intracellular insulin content + secreted insulin)). *p < 0.05 vs negative control. Values are shown as the mean ± s.e.m (n = 4). [Ca2 +]*i* levels of INS-1D cells exposed to 5% D8CM for 24 h were measured after treatment with G25 (**C**) or K30 (**D**).

To study the effects of adipocyte-derived factors on pancreatic β-cell function, we prepared conditioned-medium from the culture supernatants of the mouse 3T3-L1 cell-line (D8CM), which corresponds to the stage of mature adipocytes. When INS-1D cells were exposed to 5%, 10%, and 20% D8CM, insulin secretion stimulated by 25 mM glucose and 30 mM KCl was significantly decreased in a dose-dependent manner compared to control (Fig. 1A). In INS-1D cells exposed to 5% D8CM for 3 to 48 h, glucose stimulated-insulin secretion was decreased in an exposure time-dependent manner (Fig. 1B). In addition, after exposure of INS-1D cells to D8CM, the intracellular calcium levels ([Ca^2+^]*i*) in the presence of 25 mM glucose, measured by the fluorescent intensity of Fluo4-AM, was significantly decreased (Fig. 1C), while that in the presence of 30 mM KCl was slightly increased (Fig. 1D).

Next, the WST-1 assay was used to investigate whether exposure to D8CM affected INS-1D cell viability. As a positive control, exposure of INS-1D cells at 24 h to the oxidative stress inducer, menadione, significantly reduced cell viability in a concentration-dependent manner (Fig. S1A). D8CM had no effect on cell viability after 12 h of exposure. However, exposure to D8CM for 24 and 48 h significantly reduced cell viability in a D8CM concentration-dependent manner (Fig. S1B). Based on these results, the exposure time of D8CM in subsequent experiments was set at 24 h.

### NECAB1 is a novel regulator of insulin secretion

To elucidate the molecular mechanisms of impaired insulin secretion in D8CM-exposed INS-1D cells, we analyzed changes in gene expression profiles by DNA microarrays. As a result, D8CM up- and downregulated the expression of 1207 and 1342 genes in INS-1D cells, respectively (Table S1, 2). Although glucose-stimulated insulin secretion was reduced, the expression of molecules known to be involved in insulin exocytosis did not seem significantly changed (Table S1, 2 and Fig. S1C). Among the differentially expressed genes, we focused on *Necab1*–which was markedly increased in INS-1D cells exposed to D8CM–because it contains an EF-hand and is considered to be involved in calcium signaling, which is important for insulin secretion (Table S1, 2). NECAB1 is expressed in the hippocampus and cerebral cortex of the brain and is involved in pain signaling in the dorsal root ganglion (DRG) neurons and the spinal cord^13^. However, the expression and function of NECAB1 outside the nervous system has not been reported.

**Figure 2:**
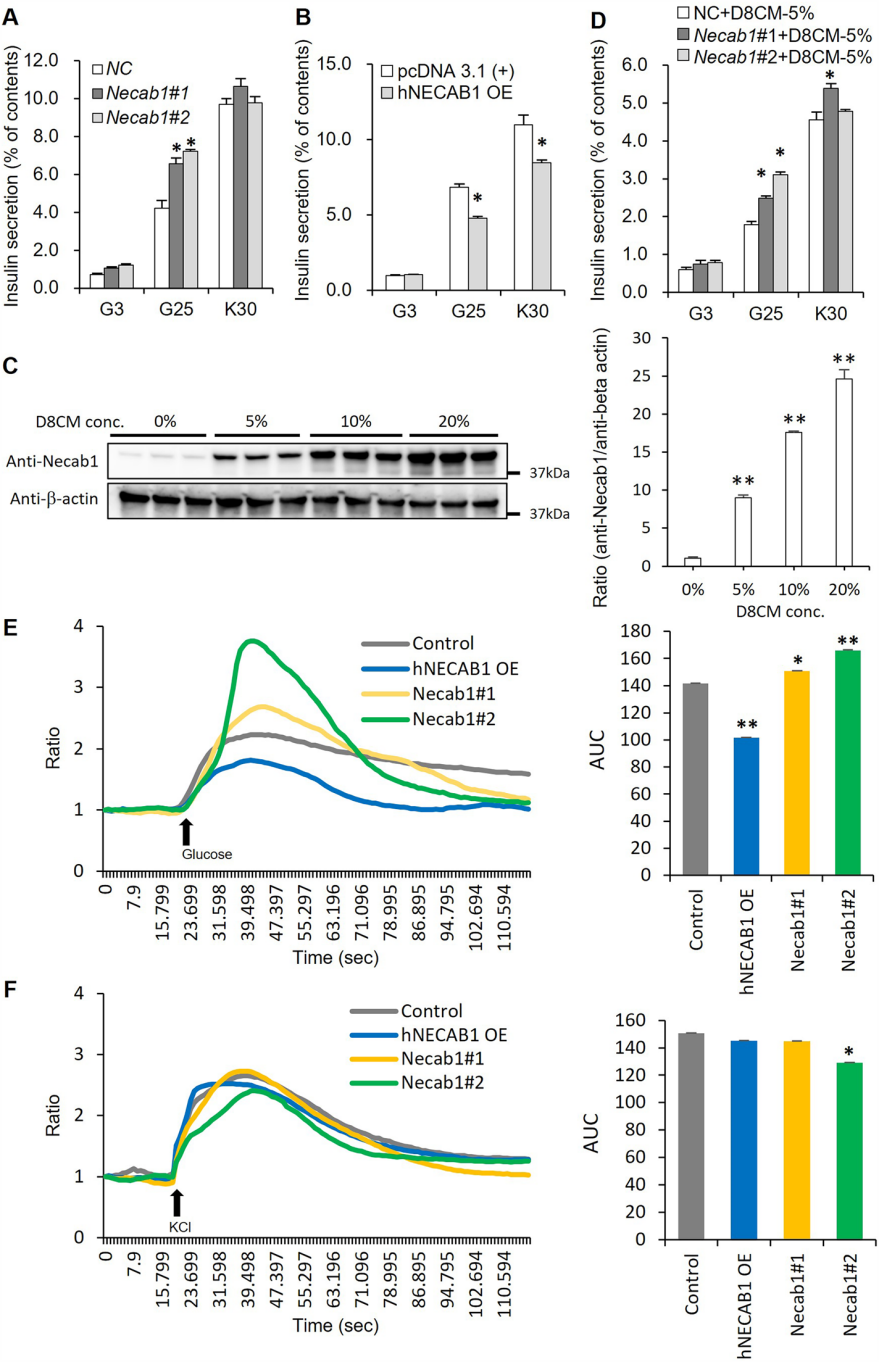
*Necab1* is induced following exposure of INS-1D cells to D8CM and is a novel negative regulator of insulin secretion. (**A**) Insulin secretion from INS-1D cells transfected with *Necab1* siRNA and Negative control siRNA (NC) after 72 h was measured after treating with 3 mM glucose (G3) or 25 mM glucose (G25) or 30 mM KCl (K30). *p < 0.05 vs NC specific silencing was confirmed by at least three independent experiments. The insulin secretion is shown as percent of insulin content (secreted insulin/(intracellular insulin content + secreted insulin)). (**B**) Insulin secretion from INS-1D cells transfected with blank vector or overexpression (OE) of human *NECAB1* vector was measured after treating with G3 or G25 or K30. *p < 0.05 vs pcDNA 3.1(+). (**C**) The expression of NECAB1 protein levels in INS-1D cells exposed to D8CM was examined by western blotting. Quantitative data are represented as the mean ± s.e.m in bar graphs. Original blots are presented in Fig. S10. (**D**) After *Necab1* knockdown, insulin secretion from INS-1D cells exposed to 5% D8CM was measured after treating with G3 or G25 or K30. *p < 0.05 vs NC + D8CM-5%. (**E,F**) The levels of [Ca2 +]*i* in INS-1D cells transfected with *Necab1* siRNA #1, #2 or with the human NECAB1 vector were measured after treatment with G25 or K30. The response of Fluo-4 was measured as area under the curve (AUC) and shown as a bar graph.

We confirmed by qPCR the expression of *Necab1* mRNA in INS-1D cells. When *Neacb1* mRNA was suppressed in INS-1D cells by transfecting siRNAs (Fig. S2A), insulin secretion was increased in the presence of 25 mM glucose, but not by KCl stimulation, when compared with the control cells (Fig. 2A). When human *NECAB1* was overexpressed in INS-1D cells, NECAB1 protein was localized in the cytoplasm (Fig. S2B), and insulin secretion was significantly decreased in the cells induced with 25 mM glucose or 30 mM KCl compared to control cells (Fig. 2B). The protein levels of NECAB1 during overexpression were comparable to those of NECAB1 at 20% D8CM exposure (Fig. S2C). These results raised the possibility that NECAB1 is a novel negative regulator of insulin secretion in INS-1D cells and that part of the decrease in insulin secretion by D8CM may involve the upregulation of NECAB1. Next, we examined the relationship between *Necab1* expression and D8CM exposure. As a result, the levels of *Necab1* mRNA and protein were significantly increased following exposure to D8CM and the effect was dose-dependent (Figs. S2D, 2C). Using immunofluorescence staining methods, NECAB1 was detected in the cytoplasm after D8CM exposure and was found to be co-localized with insulin (Fig. S2E). Furthermore, the reduction of insulin secretion in INS-1D cells after exposure to D8CM was inhibited by *Necab1* knockdown (Fig. S2F, Fig. 2D).

### Regulation of insulin secretion by *Necab1*

We investigated the effects of changes in NECAB1 expression on [Ca^2+^]*i* levels. The [Ca^2+^]*i* level during the glucose stimulation of INS-1D cells was significantly reduced by the overexpression of NECAB1 and significantly increased by the knockdown (KD) of *Necab1* (Fig. 2E). Changes in [Ca2+]*i* level during KCl stimulation were significantly reduced in *Necab1* KD #2, but not in *Necab1* KD #1 or overexpressed INS-1D cells (Fig. 2F). These results suggest that the D8CM-induced reduction in insulin secretion is partly due to changes in [Ca2 +]*i* levels caused by the increased expression of NECAB1. Next, we investigated which molecules NECAB1 interacts with in INS-1D cells. Overexpression of hNECAB1 in INS-1D cells and immunoprecipitation with NECAB1 antibody resulted in low immunoprecipitation efficiency (Fig. S3A). Therefore, conditions such as the transfection of rat and mouse NECAB1 plasmids into INS-1D cells were investigated. As a result, highly efficient immunoprecipitates were obtained by immunoprecipitation (IP) of protein-extracted samples of INS-1D cells overexpressing the rat NECAB1 plasmid with the anti-NECAB1 antibody (Fig. S3A). Specific protein bands binding to rat NECAB1 protein were detected by silver staining of SDS-PAGE gels of these IP samples (Fig. S3B). LC–MS/MS analysis of these bands identified 6-phosphofructo-2-kinase/fructose 2,6-bisphosphatase 2 (PFKFB2) (Table S3). Immunoprecipitates with anti-NECAB1 antibodies were western blotted with anti-PFKFB2 antibodies, which confirmed binding to NECAB1 (Fig. S3A). Next, the interaction between NECAB1 and PFKFB2 during D8CM stimulation was investigated. The results showed that exposure of INS-1D cells to D8CM increased NECAB1 expression as well as NECAB1 binding to PFKFB2 in a D8CM concentration-dependent manner (Fig. S3C). Reduced expression or inhibition of PFKFB2 function has been reported to reduce insulin secretion^14^. These results suggest that increased levels of NECAB1 in INS-1D cells may bind to and inhibit the function of PFKFB2.

### NECAB1 expression and insulin secretory impairment are mediated by the activation of the GR

We examined factors in D8CM that may be responsible for decreased insulin secretion and/or increased *Necab1* expression in INS-1D cells. As shown in Fig. S4A, D8CM was treated with proteolytic processing or was fractionated. After treatment with heat (98 °C for 20 min) or ProK, D8CM was still able to suppress insulin secretion when added to INS-1D cells (Fig. S4B), suggesting that the factors were resistant to protein denaturation and proteolysis. When D8CM was fractionated into WSF and ESF and the fractions were added to INS-1D cells, the lowering effect on insulin secretion was only observed with ESF but not with WSF (Fig. S4C). Consistent with this finding, the induction of *Necab1* mRNA expression was observed only in response to ESF (Fig. S4D). These results suggest that factors in D8CM mediate the increase in *Necab1* mRNA expression, and the lowering-effect of insulin secretion in INS-1D cells was attributed to non-protein and ether-soluble components.

**Figure 3:**
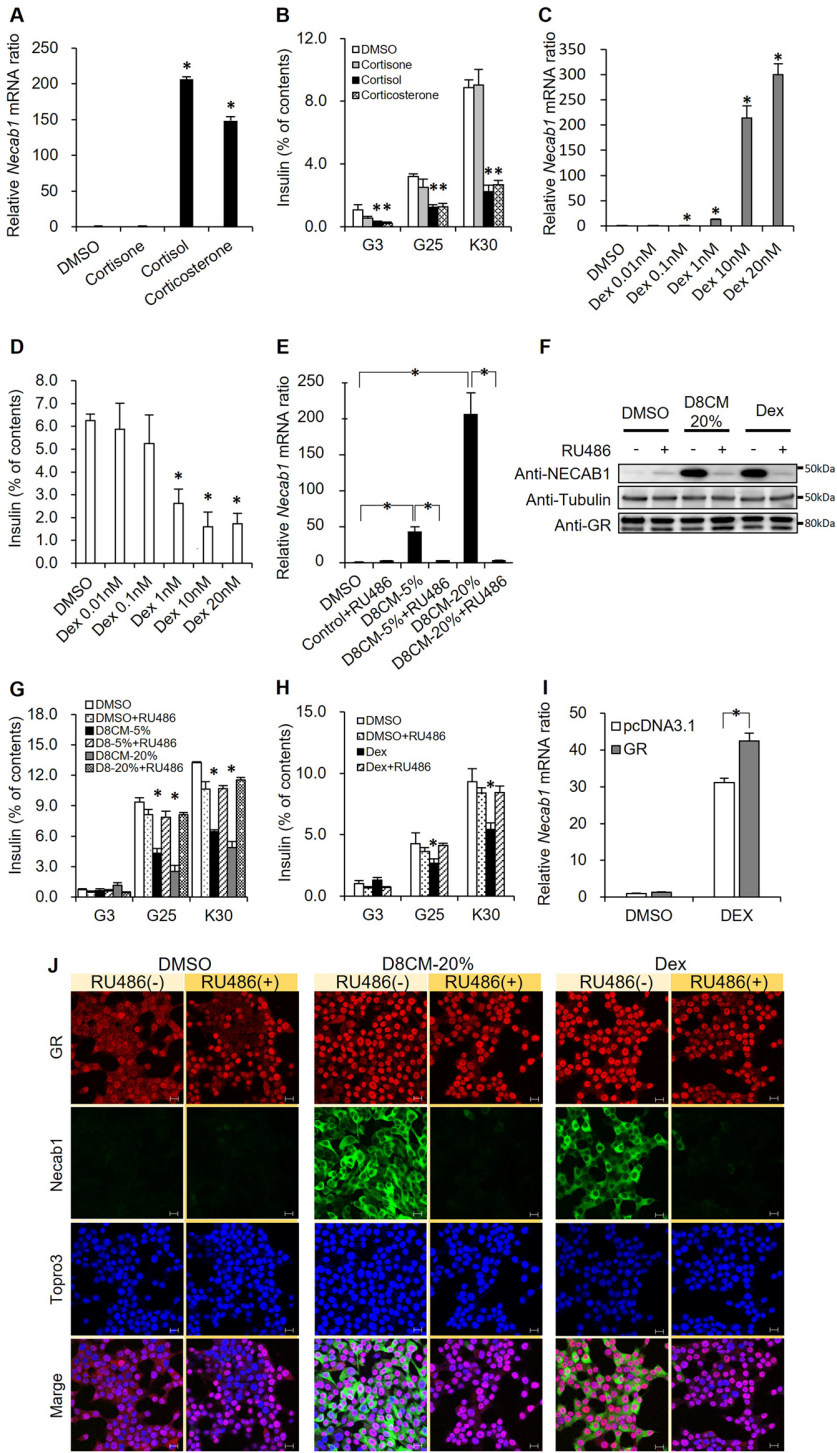
D8CM-induced Necab1 expression is dependent on GR activation in INS-1D cells. (**A**) The expression of *Necab1* mRNA levels in INS-1D cells exposed to 0.5 μΜ steroid hormone was examined by quantitative RT-PCR. Values are shown as mean ± s.e.m (n = 4). (**B**) Insulin secretion from INS-1D cells exposed to 0.5 μΜ steroid hormones for 24 h was measured after treatment with 3 mM glucose (G3) or 25 mM glucose (G25) or 30 mM KCl (K30). (**C**) The expression of *Necab1* mRNA levels in INS-1D cells treated with dexamethasone (Dex) at 0.01–20 nM was examined using quantitative RT-PCR (n = 4). (**D**) Insulin secretion from INS-1D cells exposed to Dex at 0.01–20 nM was measured using ELISA after treatment with 25 mM glucose. Insulin secretion is shown as percent of insulin content (secreted insulin/(intracellular insulin content + secreted insulin)). *p < 0.05 vs Control. (**E**) The expression of *Necab1* mRNA levels in INS-1D cells exposed to 5% and 20% D8CM and/or 1 μΜ RU-486, a GR antagonist, were examined using qPCR. (**F**) The expression of NECAB1 protein in INS-1D cells exposed to 20% D8CM and 10 nM Dex with/without RU-486, were examined using western blotting. Original blots are presented in Fig. S11. Representative data of at least three independent experiments are shown. (**G**) Insulin secretion from INS-1D cells exposed to 5% and 20% D8CM and/or 1 μΜ RU-486, for 24 h was measured after treatment with 3 mM glucose, 25 mM glucose or 30 mM KCl. *p < 0.05 vs Control. Values are shown as the mean ± s.e.m (n = 4). (**H**) Insulin secretion from INS-1D cells exposed to 1 nM Dex and/or RU-486 was measured after treatment with G3 or G25 or K30. *p < 0.05 vs DMSO. Values are shown as the mean ± s.e.m (n = 4). (**I**) Expression of *Necab1* mRNA levels in GR overexpression INS-1D cells treated with 1 nM Dex were examined using qPCR (n = 3). (**J**) NECAB1 was immunostained to detect its expression (green) using anti-NECAB1 antibodies and FITC, with insulin (Red) and nuclei (TOPRO3 blue) in INS-1D cells exposed to D8CM-20%, 10 nM Dex, and/or 1 μΜ RU-486, for 24 h. *p < 0.05 vs DMSO. Scale bar = 10 μm.

To further explore the factors that can regulate the expression of *Necab1*, we added various candidate substances to INS-1D cells (Table S4). Among them, we found that exposure to cortisol and corticosterone–both lipid-soluble–significantly increased the expression of *Necab1* mRNA and decreased insulin secretion (Fig. 3A, B). Consistent with this finding, by exposing the GR agonist Dex to INS-1D cells, both the increase in *Necab1* mRNA expression and the reduction of insulin secretion were observed in a dose-dependent manner (Fig. 3C, D). We measured the quantity of cortisol, corticosterone, and cortisone in the medium by LC–MS/MS, and found that cortisol and corticosterone, but not cortisone, were present in high levels in D8CM compared to the control-medium (Fig. S5). These data suggested that GR activation may be responsible for the D8CM-induced NECAB1 expression and impaired insulin secretion.

We examined the effects of RU-486, a GR antagonist, in our model. The increase in expression of NECAB1 and reduction in insulin secretion in INS-1D cells exposed to 5% or 20% D8CM was completely abolished by the addition of RU-486 (Fig. 3E–G). Similarly, the Dex-induced increase in NECAB1 protein expression and reduction in insulin secretion in INS-1D cells was completely negated by the addition of RU-486 (Fig. 3F, H). The Dex-stimulated expression of *Necab1* was further enhanced by overexpression of GR (Fig. 3I). Using immunostaining, we observed nuclear translocation of GR and induction of NECAB1 protein expression in INS-1D cells exposed to either D8CM or Dex (Fig. 3J). These results strongly suggest that the induction of NECAB1 expression and impairment of insulin secretion after exposure to D8CM were mediated by GR activation.

### NECAB1 is expressed in pancreatic β-cells in an obese and diabetic mouse model

To further investigate whether the suppression of insulin secretion by D8CM is reproducible in mice, we examined insulin secretion from islets in C57BL/6J mice. Insulin secretion from islets exposed to 20% D8CM was slightly decreased in the presence of 16.7 mM glucose but was significantly decreased by KCl stimulation (Fig. S6A). Insulin secretion from islets in the presence of 16.7 mM glucose or 60 mM KCl tended to decrease when exposed to 0.5 μΜ cortisol (Fig. S6B). Insulin secretion from islets exposed to 10 μΜ Dex for 24 h decreased in response to 16.7 mM glucose and 60 mM KCl (Fig. S6C). The reduction in insulin secretion from islets after exposure to 20% D8CM or 0.5 μΜ cortisol or 10 μΜ Dex for 24 h was completely negated by the addition of RU-486 (Fig. S6A–C).

Next, we examined the expression of NECAB1 in mouse islets. When the isolated pancreatic islets from C57/BL6J mice were exposed to 20% D8CM (Fig. S6D), 0.1 μΜ cortisol (Fig. S6E) and 100 nM Dex (Fig. S6F) for 24 h, the expression of *Necab1* mRNA did not change. There was no difference in the expression of *Insulin 2* mRNA at this time (Fig. S6G–I). These data indicate that although D8CM as well as cortisol or Dex suppressed insulin secretion in isolated mouse islets, it failed to increase NECAB1 expression in vitro.

**Figure 4:**
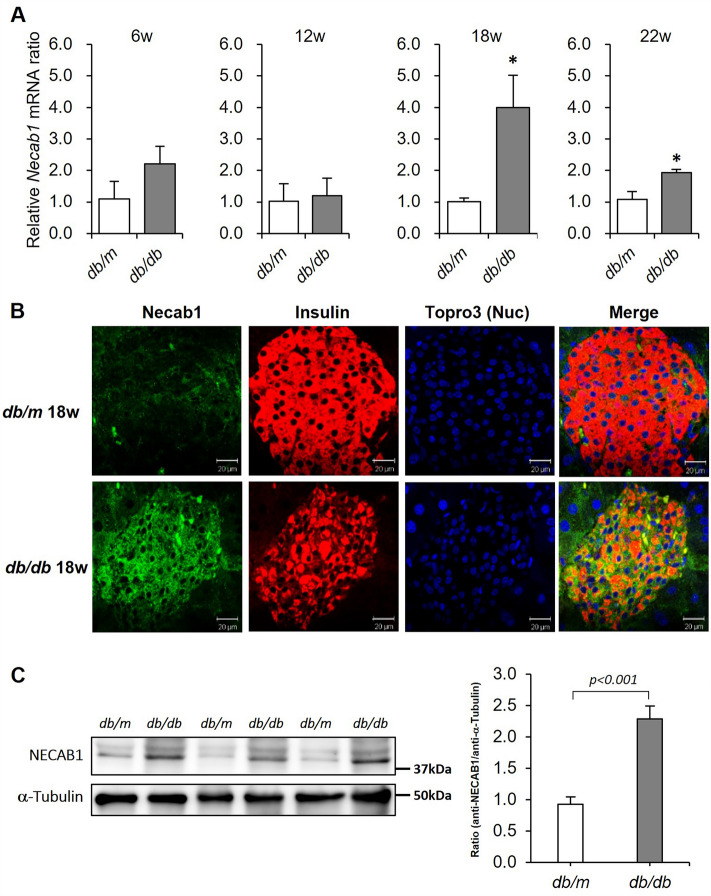
NECAB1 expression is increased in the islets of obese-diabetic model mice. (**A**) The expression of *Necab1* mRNA levels in *db/db* mice islets at 6, 12, 18, 22 weeks-old (w) was examined by quantitative RT-PCR. Values are shown as the mean ± s.e.m (n = 4). *p < 0.05 vs *db/m*. (**B**) NECAB1 was immunostained to detect its expression (green) using anti-NECAB1 antibodies and FITC, with insulin (Red) and nuclei (TOPRO3 blue) in 18-week-old *db/db* mice. (**C**) The expression of NECAB1 protein in islets isolated from 18-week-old mice were examined using western blotting. Original blots are presented in Fig. S12. Quantitative data are represented as the mean ± s.e.m in bar graphs. The immunostaining image of NECAB1 in *db/m* and *db/db* 18-week-old mice is shown in Fig. S5D. Scale bar = 20 μm. Immunostaining was confirmed by at least three independent experiments.

Thereafter, we examined the expression of NECAB1 in the pancreatic islets in an obese-diabetic mouse model* (db/db* mice). The expression of *Necab1* was analyzed by qPCR and immunostaining in *db/db* mice at 6, 12, 18 and 22 weeks. Non-fasting blood glucose levels were significantly higher in *db/db* mice compared to control *db/m* mice (Fig. S7A). Serum insulin concentrations in *db/db* mice were higher although markedly decreased between 12 and 18 weeks (Fig. S7B), and *Insulin 2* mRNA concentrations in isolated islets were significantly lower in the *db/db* mice at 18 weeks old (Fig. S7C). The expression of *Necab1* mRNA increased significantly in the islets of *db/db* mice at 18 and 22 weeks old, but not in *db/m* mice (Fig. 4A). Consistent with these results, immunostaining studies revealed that the expression of NECAB1 increased in pancreatic islets of *db/db* mice at 18 weeks compared to the control (Fig. 4B and Fig. S8A, C). In addition, a significant increase in NECAB1 protein expression in islets from *db/db* mice compared to *db/m* mice was observed by western blotting with the NECAB1 antibody in islets isolated from 18-week-old mice (Fig. 4C). As indicated by the white arrows in Fig. S8B, NECAB1 was not expressed in cells with strong insulin expression. These results suggest that NECAB1 is up-regulated in cells with reduced insulin expression in obesity and diabetes mouse models.

### Expression of *Necab1* is regulated by the binding of GR to GRE in the putative enhancer region

**Figure 5:**
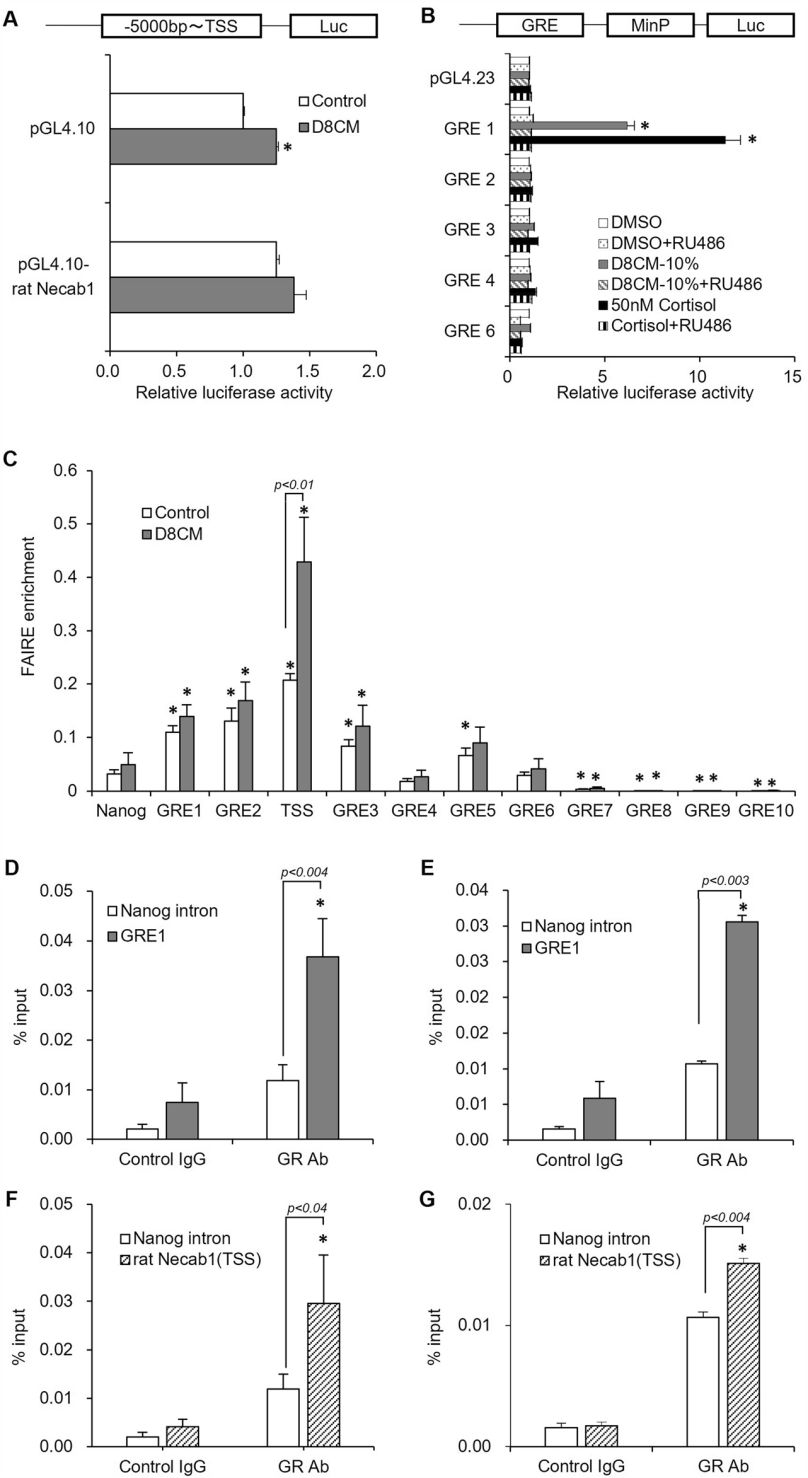
GR binds to GRE upstream of the *Necab1* gene with open chromatin in INS-1D cells. (**A**) Luciferase (luc) activity was analyzed in INS-1D cells transfected with a luc vector containing a putative promoter region of 5000 bp from *Necab1* TSS. The luc activity in INS-1D cells exposed to 20% D8CM was examined by a luc assay. *p < 0.05 vs Control. (**B**) Luc activity was analyzed in INS-1D cells transfected with luc-reporter constructs containing minimal promoter (MinP) under a GRE 1, 2, 3, 4 or 6. *p < 0.05 vs DMSO. The luc activity in INS-1D cells exposed to 10% D8CM, 50 nM cortisol and/or 1 μΜ RU-486, GR antagonist, was examined using a luc assay. *p < 0.05 vs DMSO. (**C**) Chromatin accessibility was determined by FAIRE-qPCR in GRE of the *Necab1* putative enhancer. *p < 0.05 vs Nanog Control. (**D**, **E**) Binding of GR to GRE1 of the *Necab1* putative enhancer region in INS-1D cells exposed to Dex (n = 7) and 20% D8CM (n = 3) was analyzed via a ChIP-qPCR assay. *p < 0.05 vs Control IgG. Values are shown as the mean ± s.e.m. (**F**, **G**) Binding of GR to TSS of the *Necab1* putative promoter region in INS-1D cells exposed to 100 nM Dex (n = 7) and 20% D8CM (n = 3) was analyzed via a ChIP-qPCR assay. * p < 0.05 vs Control IgG. Values are shown as the mean ± s.e.m.

Finally, we investigated the genomic region responsible for the D8CM-mediated expression of *Necab1*. Although we first created a luc-vector containing a putative promoter region spanning the 5000 bp region upstream from the rat *Necab1* TSS, D8CM did not induce a significant increase in promotor activity in INS-1D cells (Fig. 5A). Since D8CM-induced *Necab1* expression is mediated by GR activation, as we demonstrated previously, we searched for the putative GRE between the upstream 200 kbp and downstream 230 kbp in the rat *Necab1* gene. As a result, 10 putative GREs (scored > 95% concordance with the consensus sequence) were identified, and these were designated GRE 1 to GRE 10 according to the order in the rat genome (Fig. S9); GRE 1 and GRE 2 are 42.3 kbp and 33.4 kbp upstream of the rat *Necab1* TSS, respectively, and the other GREs are located in introns. The sequence of GREs 4, 5, 7, 8 and 9 were identical (Table S5).

We created luc-reporter constructs containing minimal promoter (MinP) under GRE 1, 2, 3, 4 and 6 and transfected them into INS-1D cells. As shown in Fig. 5B, GRE 1 exhibited strong reporter activity following the addition of D8CM or cortisol (6.17-fold and 11.35-fold increase when compared with the baseline, respectively), and this enhancement was negated by RU-486. Thereafter we determined chromatin accessibility by FAIRE-qPCR in GREs in the *Necab1* gene area in INS-1D cells, as well as within the region close to the TSS, to confirm whether open chromatin is induced by stimulation with D8CM. Chromatin accessibility in the basal state was shown to be higher in GRE 1, 2, 3, 5 and within the region designated as TSS (310–400 bp upstream from TSS) compared to the negative controls (*Nanog* intron region; Fig. 5C), however, this was not observed in GRE 4 and GRE 6–10. After exposure to 10% D8CM, chromatin accessibility was only significantly increased in the TSS.

We next examined GR binding to GRE 1 (high transcriptional activity and open chromatin state) and to the TSS region by ChIP-qPCR using a GR antibody in INS-1D cells transfected with a GR expression vector. We found that GR binding to GRE 1 was significantly increased following exposure to Dex (Fig. 5D) or 20% D8CM (Fig. 5E) relative to the *Nanog* intron or IgG control. We also demonstrated that both Dex (Fig. 5F) and D8CM (Fig. 5G) enhanced the binding of GR in the TSS region. Together with the fact that the 5000 bp putative promoter region containing the TSS did not increase transcriptional activity after D8CM exposure (Fig. 5A), it was suggested that GR binding to both the TSS and GRE 1 as a putative enhancer are required for the expression of *Necab1* mediated by GR activation.

## Discussion

In the present study, we examined the dysfunction of pancreatic β cells exposed to adipocyte culture supernatant (namely D8CM) and identified NECAB1 as a novel negative regulator of insulin secretion in β cells. NECAB1 is a calcium-binding protein with two N-terminal EF-hand motifs. It was originally described as one of the interacting targets for the presynaptic calcium sensor, synaptotagmin I, as well as a downstream target of Pax6 which is involved in the development of mammalian retinal primordium^15–17^. NECAB1 expression was also found in the temporal lobe of the human brain^18^. A recent study reported that NECAB1 mRNA + neurons are scattered throughout the mouse hippocampus, and NECAB1 proteins are highly enriched in mouse DRG neurons^19^. Moreover, in the spinal dorsal horn, NECAB1 and 2 are localized to glutamatergic excitatory interneurons. However, the relationship between NECAB1 and insulin secretion in pancreatic β-cells has not been reported. In this study, we identified NECAB1 for the first time as a novel negative regulator of insulin secretion in pancreatic β-cells. Since NECAB1 is a calcium-binding protein with two N-terminal EF-hand motifs, we examined the association between the increase in NECAB1 expression and the decrease in calcium influx following exposure to D8CM. We found that changes in NECAB1 expression alter calcium signaling and identified PFKFB2 as a molecule that binds to the upregulated NECAB1. Altered expression of PFKFB2 is known to be involved in insulin secretion^14^. Merrins et al*.* reported that changes in PFKFB2 levels alter Ca^2+^ oscillations in islets, irrespective of GK activity^20^. It was suggested that NECAB1 may inhibit PFKFB2 function by binding to PFKFB2, thereby reducing Ca^2+^ oscillation and insulin secretion. However, future studies are needed to clarify the precise mechanisms by which NECAB1 binding to PFKFB2 suppresses insulin secretion.

Glucocorticoids have a variety of biological roles, including metabolic regulation. It has been well established that excess glucocorticoids give rise to insulin resistance, leading to diabetes mellitus both in humans and rodents^21–23^. It has also been reported that glucocorticoid directly suppresses insulin secretion in cultured β-cells^24^ and mouse islets in vivo^25–27^. Transgenic mice expressing glucocorticoid receptors in pancreatic β-cells exhibited impaired insulin secretion^28,29^. Recently, Burke et al. reported that glucocorticoids impaired islet β-cell function via GR activation^30^.

Alberts et al. reported that circulating glucocorticoid concentrations were higher in the *ob/ob* mouse compared to the normal C57BL/6J mouse^31^. The glucocorticoid concentrations of hepatic, mesenteric and epididymal adipose tissue are also higher in the *ob/ob* mouse compared to the normal C57BL/6J mouse. It has been shown that the serum corticosterone concentration in *db/db* mice was markedly increased, and blood glucose was reduced following treatment with RU-486^32^. In in vitro reports, direct exposure of β-cells to glucocorticoids results in altered β-cell function, and reduced glucose sensitivity and insulin secretion^33^. An in vivo study reported that short-term GC treatment causes hyperinsulinemia and increased GSIS because of the compensatory action of pancreatic β-cells against insulin resistance^34,35^. As described above, various studies have reported direct effects of glucocorticoids on β-cells, showing different results. The differences may be due to HSD11B1 expression and GR activity in adipose tissue and pancreatic β-cells, host pathological context such as pregnancy or obesity, and experimental systems such as in vivo and in vitro^33^.

In agreement with our data, Zawalich et al. reported that RU-486 recovered insulin secretion from Dex-treated isolated pancreatic islets in rats^36^. These lines of evidence support the idea that the mechanism of GR-regulated insulin secretion is conserved. Since D8CM-induced impairment of insulin secretion was mainly mediated by GR activation, our system may be a useful model to analyze pancreatic β-cell dysfunction associated with an increase in glucocorticoid production in obesity.

The upstream region of the *Necab1* gene is highly variable among species. In humans and mice, the same sequence as rat GRE 1 was present upstream at 44.7 kbp and 17.7 kbp respectively. It is possible that the regulation of NECAB1 expression by GR may be different among species or be context dependent.

Our findings may also have clinical implications for diabetes. We found that the expression of NECAB1 was increased in the islets of 18-week-old diabetic-obese model mice, which corresponded with the period when insulin deficiency progressively developed. We do not know, however, why the expression was induced in pancreatic islets within such a specific time frame. Furthermore, differential expression of NECAB1 in isolated islets and INS-1D cells was observed in response to glucocorticoids. NECAB1 has been shown to be elevated in islets in pathological conditions such as diabetes and obesity. Therefore, the promotion of GR activation in normal islets alone may not be a prerequisite for an increase in NECAB1 expression. More detailed analysis, using genetically modified mice, is necessary to elucidate the precise role of NECAB1 in pancreatic islets in the normal state or development of diabetes mellitus.

In humans, Chalew et al*.* revealed that plasma cortisol levels increase with age in obese subjects^37^. It was also recently reported that high serum cortisol levels were correlated with decreased insulin secretion in a general Japanese population^38^. While insulin secretion was enhanced in typical cases of Cushing's syndrome and steroid-induced diabetes, it is plausible to speculate that GR-regulated NECAB1 expression in β-cells may have a pathophysiological role in diabetes development in some stages of the disease.

In conclusion, we identified NECAB1 as a negative regulator of insulin secretion, and induction of NECAB1via GR activation may mediate pancreatic β-cell dysfunction in some contexts such as obesity-related diabetes mellitus. Future studies may prove targeting GR and/or NECAB1 in β-cells as a novel therapeutic approach for obesity-related diabetes mellitus, which has become a worldwide pandemic.

## Methods

### Cell culture

INS-1D cells were generously gifted by Dr C.B. Wollheim and Dr N. Sekine. INS-1D cells were cultured at 37 °C in a 5% CO_2_-enriched atmosphere in RPMI1640 medium (Sigma-Aldrich, St. Louis, MO) with 10% heat-inactivated fetal bovine serum (Sigma-Aldrich), 50 μM 2-mercaptoethanol, 1 mM sodium pyruvate, 10 mM HEPES, 100 U/ml penicillin, and 100 μg/ml streptomycin (Invitrogen, Carlsbad, CA)^39^. 3T3-L1 cells were incubated at 37 °C in Pre-Adipocyte Medium (ZEN-BIO, INC.). Two days after the cells reached confluency, they were cultured for three days in Differentiation Medium (ZEN-BIO, INC.). Thereafter the medium was replaced with Adipocyte-Medium (ZEN-BIO, INC.). After seven days, cells were washed twice with RPMI medium, and INS-1D medium was added to produce 3T3-L1 cell CM. One day later, the day eight conditioned medium (D8CM) was removed and filtered through a 0.2 μm filter and stored at -80 °C. As shown in Fig. S4, proteolysis and fractionation of D8CM were performed as follows: D8CM was heat-treated at 98 °C for 20 min; protein degradation of D8CM was achieved by treatment with Proteinase K (ProK; TAKARA BIO INC. Shiga, Japan); one volume of D8CM was combined with four volumes of ether (1:4 v/v); the two extracts were concentrated using a centrifugal concentrator (VC-36N, TAITEC Co., Ltd.); the water-soluble fraction (WSF) was dissolved in RPMI medium, and the ether soluble fraction (ESF) was dissolved in DMSO.

### Animals

This study was approved by the National Center for Global Health and Medicine research ethics committees (Approval No. 14010, 15047 and 16031). All animal studies were carried out in accordance with relevant guidelines and regulations and ARRIVE guidelines. C57BL/6J male mice and BKS.Cg-m + / + *Leprdb*/Jcl (*db/m*), BKS.Cg- + *Lepr*^*db*^*/* + *Lepr*^*db*^/Jcl (*db/db*) male mice were purchased from CLEA Japan Inc. (Tokyo, Japan).

### Plasmid constructs

A Myc-DDK tagged human, rat, and mouse N-Terminal EF-Hand Calcium Binding Protein 1 (*Necab1*) expression vector was purchased from ORIGENE Technologies Inc. (Rockville, MD; Cat# RC209704). A fragment of the rat *Necab1* putative promoter region (5000 bp upstream of rat *Necab1* transcription start site (TSS), Rat Genome Sequencing Consortium v3.4/rn4 in the NCBI Assembly database)^40^ was amplified by polymerase chain reaction (PCR) in a mixture containing INS-1D genomic DNA, *Necab1* primers (Table S6) and KOD plus neo (Toyobo, Osaka, Japan) to generate the rat *Necab1* promoter constructs. The 5000 bp putative promoter region mentioned above was cloned into the pGL4.10 vector (Promega, Madison, WI, USA). The resulting construct was named pGL4.10-rat *Necab1*. The Glucocorticoid Responsive Element (GRE) 1, 2, 3, 4, and 6 (Table S7), and GR (Table S8) were produced by ligating the corresponding double-strand oligonucleotides to the pGL4.23 vector (Promega). All constructs were verified by DNA sequencing.

### Measurement of metabolic components

Blood glucose levels were measured using a portable glucose meter (Glutest, Sanwa kagaku kenkyusyo co., LTD, Aichi Japan). Insulin levels were determined by an enzyme-linked immunosorbent assay (Morinaga Institute of Biological Science, Inc., Yokohama, Japan).

### Transfection of small interfering RNAs

Silencer select small interfering RNAs (siRNA) targeting rat *Necab1* (#1; s133845, #2; s133846), and a non-targeting negative control (NC) siRNA #5 (AM4642) were obtained from Thermo Fisher Scientific (Waltham, MA). INS-1D cells that had been passaged 25–30 times were trypsinized and re-suspended in RPMI medium without antibiotics. Various siRNAs, negative control siRNA, or vector were transiently transfected with Lipofectamine 2000 reagent (Invitrogen, Carlsbad, CA) according to the manufacturer’s instructions. Briefly, 2.5 µl of Lipofectamine 2000 was diluted in 125 µl of serum-free RPMI medium per well and pre-incubated for 5 min. Each siRNA was diluted in 125 µl of serum-free RPMI medium to a final concentration of 20 µM. The two mixtures were combined and incubated for 20 min to allow complex formation. After 20 min, 750 µl of RPMI medium containing INS-1D cells (5 × 10^5^ cells/ml) was added to the mixture and transferred to 24-well plates. This resulted in a final siRNA concentration of 10 nM. mRNA expression was assayed 48 h after transfection, and specific transcriptional silencing was confirmed by at least three independent experiments.

### Insulin secretion and content

After the indicated culture periods, INS-1D cells were washed with Krebs–Ringer HEPES (KRH) buffer with 0.1 mM glucose, pre-incubated at 37 °C for 30 min in KRH buffer containing 3 mM glucose, and incubated at 37 °C for 60 min with 3 mM and then 25 mM glucose. Insulin secreted into the KRH buffer was measured using the Mouse Insulin ELISA kit (T-Type; AKRIN-011 T, Shibayagi, Gunma, Japan). Cellular insulin was extracted with acid–ethanol overnight at 4 °C, and insulin content was determined using an ELISA after appropriate dilution.

### Intracellular calcium analysis

Intracellular calcium concentration ([Ca^2+^]*i*) in INS-1D cells was measured as previously described^41^ with a Calcium Fluo-4 kit (#CS22; Dojindo Molecular Technologies, Inc., Kumamoto, Japan). INS-1D cells were placed on a φ35 mm glass bottom culture dish, the growth medium was removed and replaced with loading buffer containing 3 mM glucose and Fluo4-AM, and the culture dish was incubated at 37 °C for 1 h. Loading buffer was removed and replaced with recording buffer. [Ca^2+^]*i* levels were detected when stimulated with 25 mM glucose and 30 mM KCl. Fluo-4 fluorescent signal over the INS-1D cell area was detected by excitation at 488 nm on an LSM 510 Live microscope (Carl Zeiss, Oberkochen, Germany).

### RNA preparation and quantitative real-time PCR (qPCR)

Total RNA was extracted from INS-1D cells or mouse pancreatic islets using the RNeasy Mini Kit (Qiagen, Cologne, Germany). cDNA was prepared from 1 μg of total RNA using reverse transcriptase (SuperScript IIITM, Invitrogen) according to the manufacturer’s instructions. Quantitative PCR amplification was performed and analyzed using the TaqMan universal PCR master mix core reagent kit on StepOnePlus (Applied Biosystems, Foster, CA). The mRNA levels were evaluated with reference to *beta actin* (*Actb*) expression using the 2ΔΔCt method^42^. TaqMan gene expression assay probes for rat *Necab1* and *Actb* were obtained from Applied Biosystems (catalogue numbers Rn00574261_m1 and 4352931E, respectively, Applied Biosystems, Foster, IN). TaqMan gene expression assay probes for mouse *Necab1*, *Actb*, *Insulin 1*, and *Insulin 2* were obtained from Applied Biosystems (Mm00724274_m1, Mm01205647_g1, Mm01278327_m1, and Mm03038438_m1, respectively).

### Microarray Analysis

RNA expression in the INS-1D cells was compared using the Rat 230 2.0 Array (Affymetrix, Santa Clara, CA, USA; n = 2) as described previously^43^. Samples for the analysis were prepared in accordance with the manufacturer's protocol, and the results were normalized using the Affymetrix operating system, GeneChip® Operating Software (GCOS), and analyzed using the Database for Annotation, Visualization, and Integrated Discovery 6.7, otherwise known as DAVID 6.7^44^.

### Cell proliferation assays

INS-1D cell proliferation was evaluated using a WST-1 assay kit (Takara Bio Inc.Shiga, Japan). Cells were seeded in 96-well plates and exposed to either D8CM or Menadione. After 24, 48 and 72 h, WST-1 solution was added and the cells were incubated for an additional 2 h, after which absorbance was measured at 450 nm.

### Simultaneous analysis of steroid hormones by LC–MS/MS

Steroid hormones in D8CM and control medium were analyzed by LC–MS/MS (ASKA Pharma Medical Co., Ltd., Kawasaki, Japan)^45^. Steroids labeled with stable isotopes were added to the sample as an internal standard, and after dilution with pure water, the material was extracted with methyl tert-butyl ether (MTBE). The organic solvent layer was dried, and the residue was dissolved in a methanol/pure water mixture.

Steroid fractions were prepared using a mixed mode reverse-phase system solid phase column (60 mg, 3 cc) Oasis MAX (Waters Co., Milford, MA). After the steroid fractions were dried, the material was dissolved in an ethanol/hydrochloric acid mixture (5:1) and allowed to react. The residue was neutralized with a sodium hydrogen carbonate solution, extracted with MTBE, and left to dry. Picolinic acid derivatization reagent (prepared by dissolving 2-methyl-6-nitrobenzoic acid anhydride, picolinic acid, and 4-dimethyl amino pyridine in acetonitrile) and triethylamine were added to the residue, mixed, and allowed to react for 30 min. The reaction solution was diluted with a hexane/ethyl acetate/acetic acid mixture, and the derivative fraction was prepared with an order phase system solid-phase column (500 mg, 3 ml) InertSep SI (GL Science, Tokyo, Japan). After drying, the material was dissolved in an acetonitrile/purified water solution and loaded onto an LC–MS/MS. A Shimadzu Nexera system was used for LC with Phenomenex Kinetex C18 (1.7 µm, 2.1 × 150 mm), Capcellcore C18 (2.6 µm, 2.1 × 150 mm), and Capcellcore ADME (2.7 µm, 2.1 × 150 mm) as a column (Shimadzu Co., Kyoto, Japan). MS/MS (AB Sciex API4000), at the particular m/z of each steroid using ESI (Positive) mode (Framingham, MA), was performed. A calibration line for each steroid was created with the peak area ratio, and the steroid concentration of each sample was determined.

### Western blotting

Proteins from INS-1D cells were extracted using NP-40 buffer, separated by electrophoresis on a 4–20% sodium dodecyl sulfate Poly-Acrylamide Gel Electrophoresis (SDS-PAGE) gradient gel, and transferred to a polyvinyldenedifluoride membrane (BioRad Laboratories, Hercules, CA). Western blot analyses were performed using anti-mouse NECAB1 (ORIGENE Technologies Inc., TA502630), anti- rabbit α-Tubulin (Cell Signaling Technology #2144), anti-GLUT2 (No. 07-1402, Millipore Co., Billerica, MA), anti-E-cadherin (BD Transduction Laboratories, Franklin Lakes, NJ, USA; #610,181), anti-KCNJ11 (No. 16920-1-AP, Proteintech Group, Inc., Rosemont, IL, USA ), anti-VAMP2 (No. AB5856, Millipore Co.), anti-HSP70 (No. PA5-28003, Invitrogen) and anti-PFKFB2 (No. TA314335, OriGene) antibodies and detected with Immobilon Western Chemiluminescent HRP Substrate (Millipore Co.).

### Immunocytochemistry

INS-1D cells were fixed with 4% (w/v) paraformaldehyde for 15 min, permeabilized with 0.25% (w/v) Triton X-100 in PBS for 15 min, and immunostained as described previously^46,47^. After blocking with PBS containing 10% (w/v) BSA for 30 min, cells were incubated overnight with the primary antibody in PBS containing 3% (w/v) BSA, washed with PBS, and incubated with secondary antibody in PBS containing 3% (w/v) BSA for 1 h, followed by washing with PBS and mounting. Immunofluorescent images were obtained using a Zeiss LSM510 confocal microscope in confocal mode.

### Immunostaining

Pancreata were excised, fixed in 4% paraformaldehyde, embedded in paraffin, and immunostained as previously described^46,47^. Anti-rabbit NECAB1 (HPA025963, Atlas Antibodies Stockholm, Sweden) and guinea pig anti-Insulin (Takara Bio Inc.) antibodies were used. FITC-conjugated anti-rabbit (1:100) (Jackson ImmunoResearch) and DyLight 594-conjugated anti-guinea pig IgG (1:100) were used as the secondary antibodies, and TOPRO3 (1:1000) was used to label the nuclei (Molecular Probes). Immunofluorescent images were obtained using a Zeiss LSM510 confocal microscope (Carl Zeiss Co., Tokyo, Japan) in confocal mode.

### Dual-luciferase assay

INS-1D cells were cultured for 24 h in 12-well plates (3 × 10^5^ cells/well)^22^. The cells were then incubated with a reaction mixture containing 1.2 μg of the reporter gene construct, 0.4 μg of pRL-TK vector (Promega), and 1.3 μl of Lipofectamine 2000 (Invitrogen). The cells were harvested 24 h later and luciferase activity was determined using a luminometer (CenrtoPRO #LB962 Berthold Technologies, GmbH & Co. KG).

### Chromatin immunoprecipitation (ChIP) assay

ChIP assay was performed by partially modifying the method described previously^48^. INS-1D cells were fixed in formaldehyde solution (final concentration, 1%) for 10 min, washed using ice-cold PBS, and collected as before. The cell lysates were sheared by sonication and the DNA was analyzed on a 2% agarose gel. The DNA–protein complexes were immunoprecipitated with 2 μg of anti-GR (#3660, Cell Signaling Technology) or Normal Rabbit IgG (#2729, Cell Signaling Technology). The purified immunoprecipitated DNA was quantified by qPCR using GRE1 specific primers and SYBR green (see Table S8).

### Formaldehyde-assisted isolation of regulatory elements (FAIRE) qPCR assay

INS-1D cells were cultured for 24 h in a φ15cm dish (1 × 10^7^ cells/well) with or without D8CM. INS-1D cells were fixed in formaldehyde solution (final concentration, 1%) for 10 min. The cells were thereafter washed with ice-cold PBS and collected. The cell lysates were sheared by sonication and the DNA was analyzed on a 1% agarose gel. The sheared DNA was isolated using two extractions with phenol/chloroform/ isoamyl alcohol and one extraction using chloroform. The isolated DNA was reverse-crosslinked and purified by ethanol precipitation. The purified DNA was quantified by qPCR^49,50^; qPCR was performed using the FAIRE-qPCR primers (see Table S9).

### Islet isolation and batch incubation

Islets of Langerhans were isolated from the pancreas of 6, 12, 18, 22-week-old C57BL/6J male mice by collagenase digestion^41,51^. Subsequently, the isolated islets were washed with 8.5% sucrose solution and then hand-picked under a microscope. Isolated pancreatic islets were exposed to 20% D8CM, 10 nM or 100 nM dexamethasone (Dex), 0.1 μM or 0.5 μM cortisol, and/or 1 μM RU-486 for 24 h in RPMI 1640 medium containing 10% fetal bovine serum. Islets were then washed and preincubated at 37 °C for 30 min in 0.1% BSA-KRH with 2.8 mM glucose^52^. The islets were incubated in the buffer containing 16.7 mM glucose and 60 mM KCl for 30 min. Cellular insulin content was extracted with acid–ethanol. Insulin that had been secreted into the KRH buffer was measured using the Ultra-Sensitive Mouse Insulin ELISA Kit (Morinaga Institute of Biological Science, Inc., Yokohama, Japan).

### Silver staining and LC–MS/MS analysis

Silver staining of SDS-PAGE gels was performed using a SilverQuest™ Silver Staining Kit (Invitrogen). Specific protein bands assumed to bind NECAB1 were analyzed by LC–MS/MS (ASKA Pharma Medical Co., Ltd., Kawasaki, Japan).

### Statistics

The data are presented as the mean ± s.e.m; statistical significance was determined using two-tailed unpaired Student’s *t*-tests (*p* < 0.05).

### Supplementary Information

Supplementary Information.

## Data Availability

The data sets supporting this study are available from the corresponding author, K.Y., on request.

## Abbreviations

T2D: Type 2 diabetes
D8CM: Day 8 conditioned medium
GR: Glucocorticoid receptor
ProK: Proteinase K
WSF: Water-soluble fraction
ESF: Ether-soluble fraction
KRH: Krebs-Ringer HEPES
siRNA: Small interfering RNA
PCR: Polymerase chain reaction
TSS: Transcription start site
GRE: Glucocorticoid Responsive Element
NC: Negative control
Necab1: N-Terminal EF-Hand Calcium Binding Protein 1
[Ca^2+^]*i*: Intracellular calcium concentration
*Actb*: Beta actin
qPCR: Quantitative real-time PCR
FAIRE: Formaldehyde-Assisted Isolation of Regulatory Elements
ChIP: Chromatin immunoprecipitation
Dex: Dexamethasone
Luc: Luciferase
