# Supplementary material for: Glucocorticoid receptor-NECAB1 axis can negatively regulate insulin secretion in pancreatic β-cells
Source: Sci Rep. 2023 Oct 20;13:17958. doi: 10.1038/s41598-023-44324-y (PMC10589354; doi:10.1038/s41598-023-44324-y)
Supplement: Supplementary file 1 — Supplementary Information. [file 41598_2023_44324_MOESM1_ESM.pdf]

Fig. S1 Udagawa

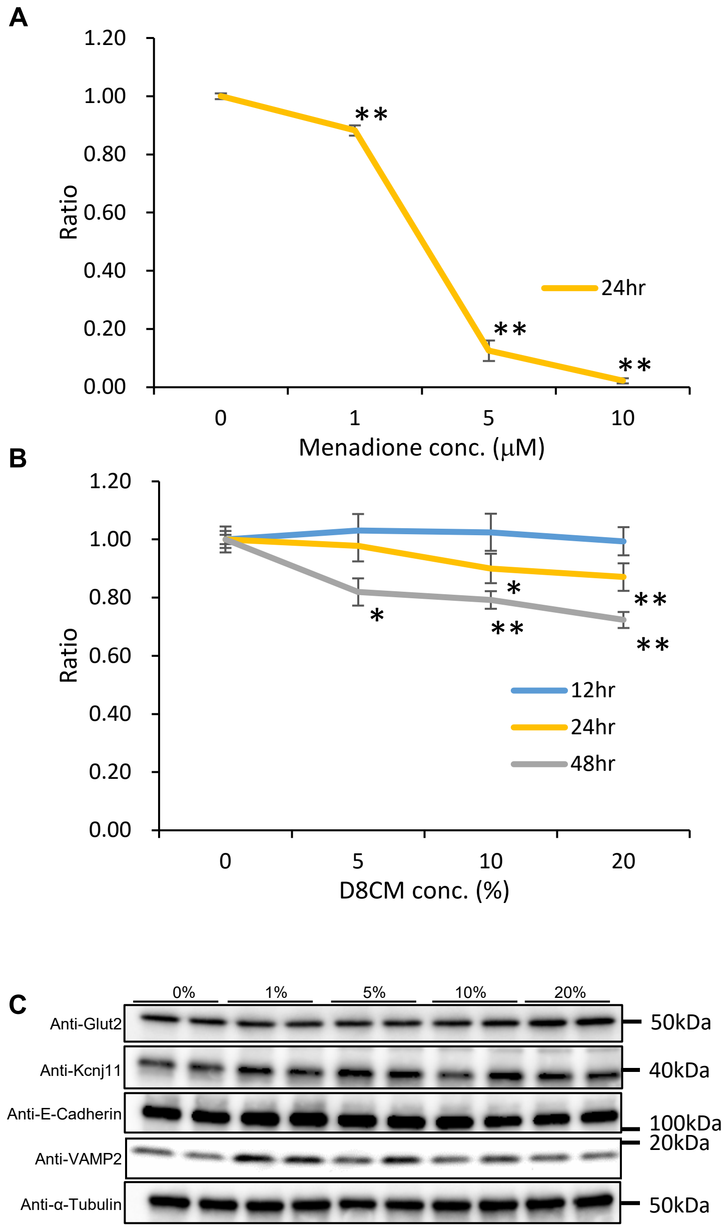

Fig. S1 Cell viability of INS1D cells.
Cells were treated with the oxidative stress inducers menadione (A) and D8CM (B) and cell viability was analysed by the WST-1 method. Values are shown as means±s.e.m. (n=3). *: p<0.05 vs Control. **: p<0.01 vs Control. (C) Glut2, Kcnj11, VAMP2 Protein levels in INS-1 cells exposure to D8CM were detected by Western Blotting. Original blots are presented in Fig. S13.

Fig. S2 Udagawa

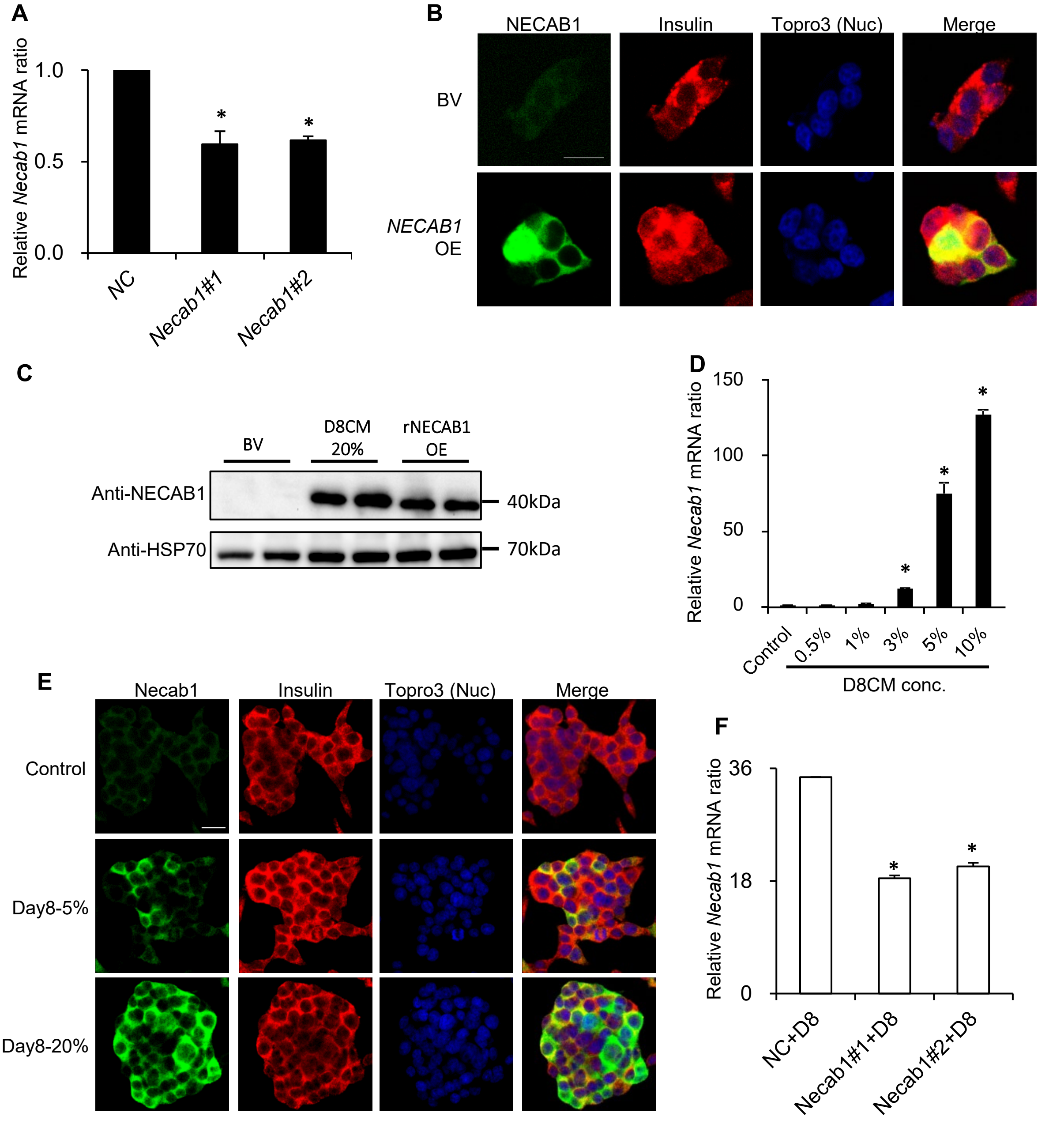

Fig. S2 Expression levels of *Necab1* mRNA in INS-1 cells

(A) The expression of *Necab1* in INS-1 cells at 48 h after transfection with siRNA was examined by qPCR. *: $p<0.05$ vs NC. Values are shown as means ± s.e.m. (n=3).

(B) Overexpression of NECAB1 and blank vector (BV) was immunostained to detect GFP (green) using anti-NECAB1 antibodies, and Insulin (Red) and nuclei (TOPRO3 blue) in INS-1 cells exposed to D8CM. Scale bar = 20 μm

(C) The expression levels of *Necab1* mRNA in INS-1 cells exposed to D8CM was examined by qPCR. *: $p<0.05$ vs Control. Values are shown as means ± s.e.m. (n=3).

(D) NECAB1 and HSP70 protein levels in INS-1 cells D8CM exposure or NECAB1 plasmid of overexpression were detected by Western Blotting. Original blots are presented in Fig. S14.

(E) Localization of endogenous NECAB1 protein was immunostained to detect GFP (green) using anti- NECAB1 antibodies, and Insulin (Red) and nuclei (TOPRO3 blue) in INS-1 cells exposed to D8CM. Scale bar = 20 μm.

(F) After *Necab1* knockdown, relative *Necab1* mRNA levels in INS-1 cells exposed to D8CM were detected by qPCR. *: $p<0.05$ vs NC. Values are shown as means ± s.e.m. (n=3). Specific silencing was confirmed by at least three independent experiments.

Fig. S3 Udagawa

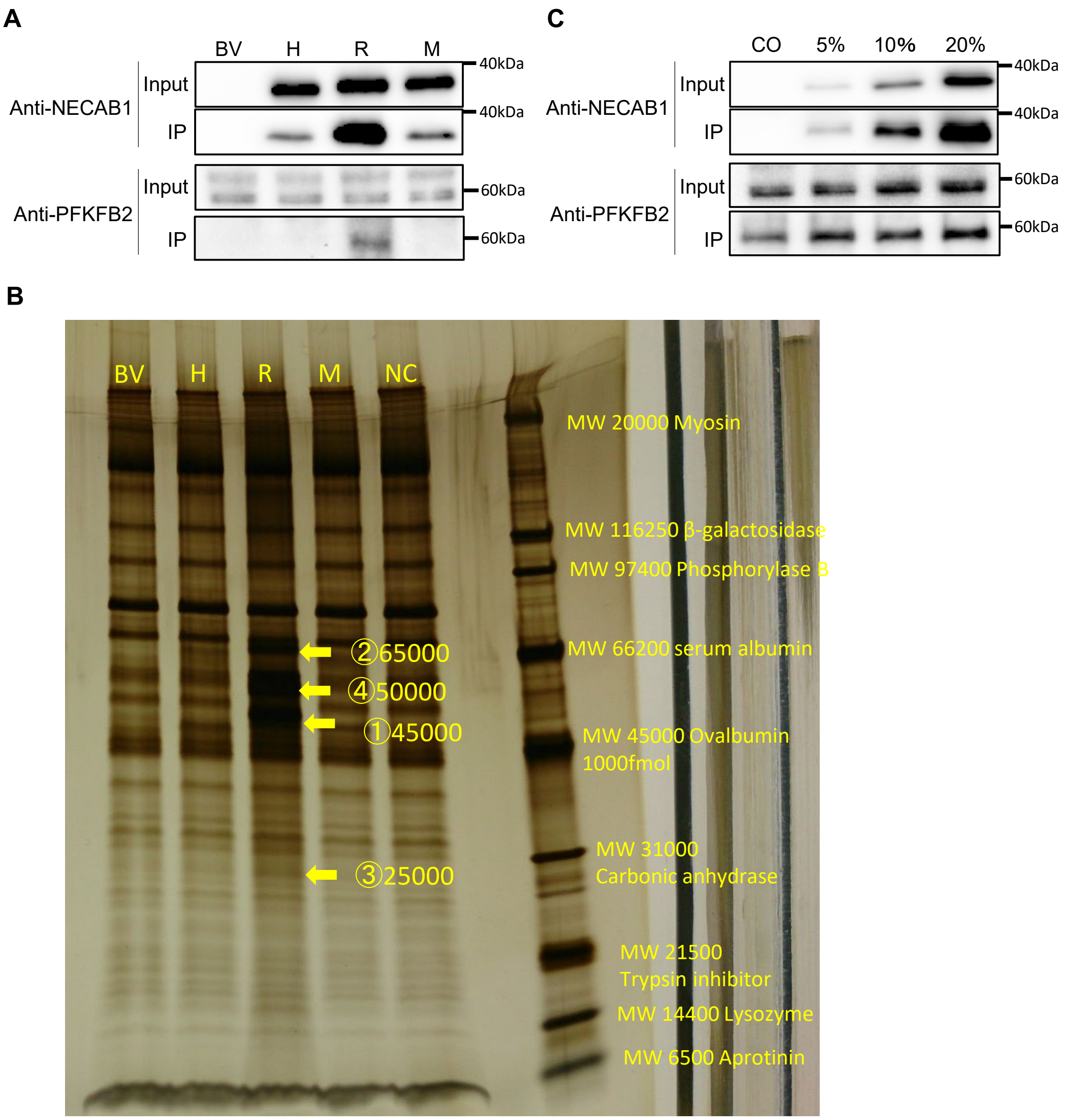

Fig. S3 Identification of proteins interacting with NECAB1 in INS-1 cells.

(A) Immunoprecipitation of NECAB1-overexpressing INS-1D cell extracts with anti-NECAB1 antibody and Western blotting with anti-PFKFB2 antibody. Original blots are presented in Fig. S15.

(B) Silver-stained images of samples overexpressing Bland vector (BV), human (H), rat (R) and mouse (M) NECAB1 and immunoprecipitated with anti-NECAB1 antibody followed by SDS-PAGE. Original blots are presented in Fig. S15.

(C) Immunoprecipitation of D8CM-exposed INS-1D cell extracts with anti-NECAB1 antibody and Western blotting with anti-PFKFB2 antibody. Original blots are presented in Fig. S16.

Fig. S4 Udagawa

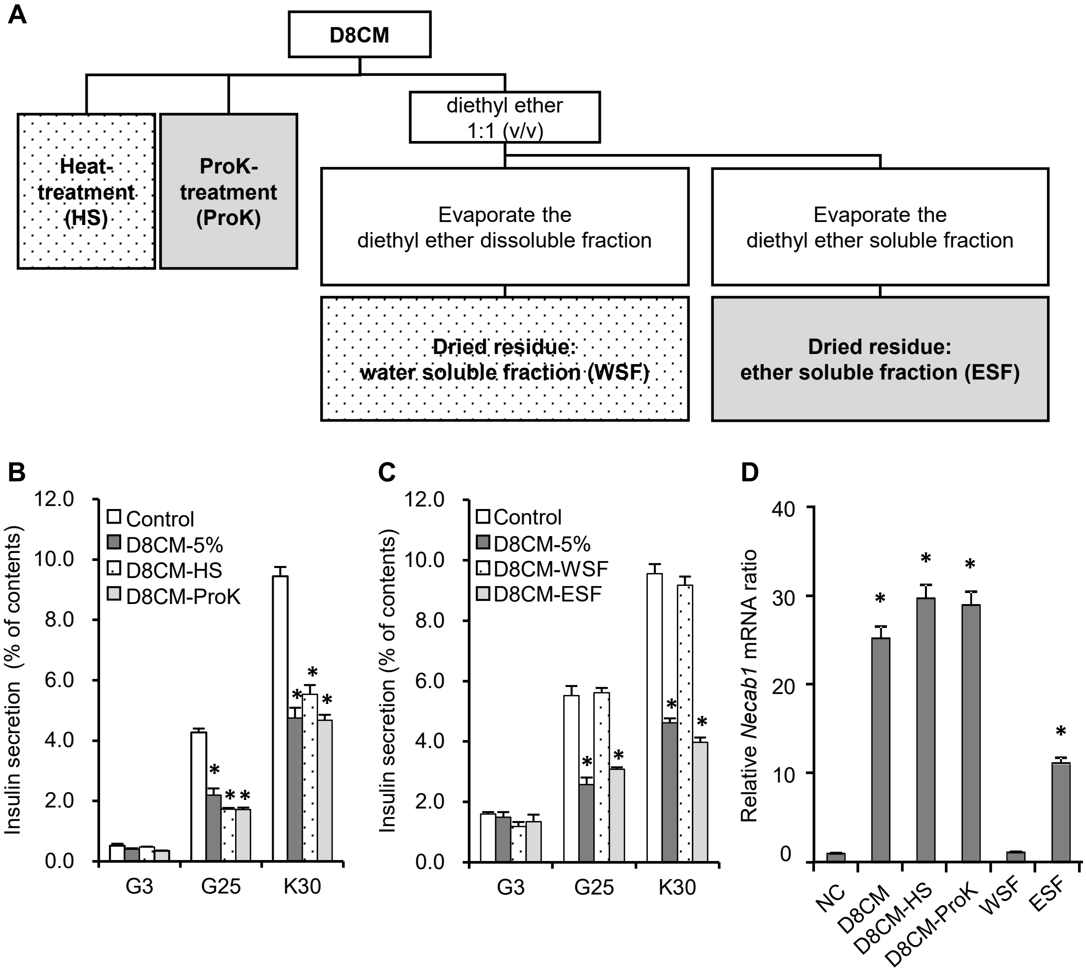

Fig. S4 Proteolysis or fractionation of D8CM

(A) Schema for the preparation of water (WSF) and ether soluble fractions (ESF), and heat (HS) and proteinase K (ProK) treatment.

(B) Insulin secretion from INS-1 cells exposed to 5% D8CM, HS, and ProK treatments D8CM for 24 h were measured after treating with 3 mM glucose (G3) or 25 mM glucose (G25) or 30 mM KCl (K30). *: p<0.05 vs Control.

(C) Insulin secretion from INS-1 cells exposed to 5% D8CM, WSF and ESF for 24 h was measured after treating with G3, G25, or K30. *: p<0.05 vs Control.

(D) Relative *Necab1* mRNA levels in INS-1 cells exposed to proteolysis D8CM or extracted D8CM, were detected by qPCR. *: p<0.05 vs NC. Values are shown as means±s.e.m. (n=4).

Fig. S5 Udagawa

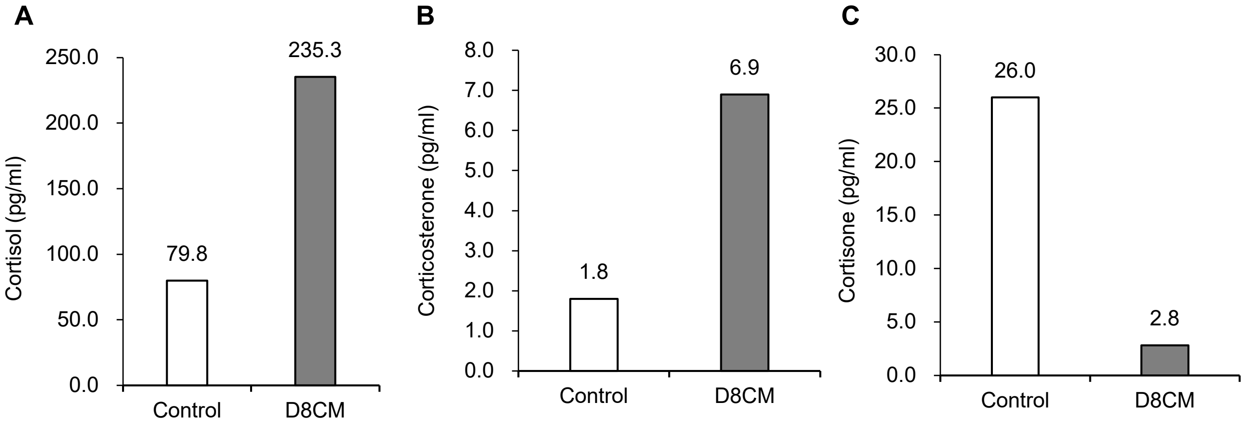

Fig. S5 Quantitative analysis of cortisol, corticosterone, and cortisone in D8CM by LC-MS/MS

D8CM and control medium with the concentration of cortisol (A), corticosterone (B) and cortisone (C) analyzed by LC-MS/MS.

Fig. S6 Udagawa

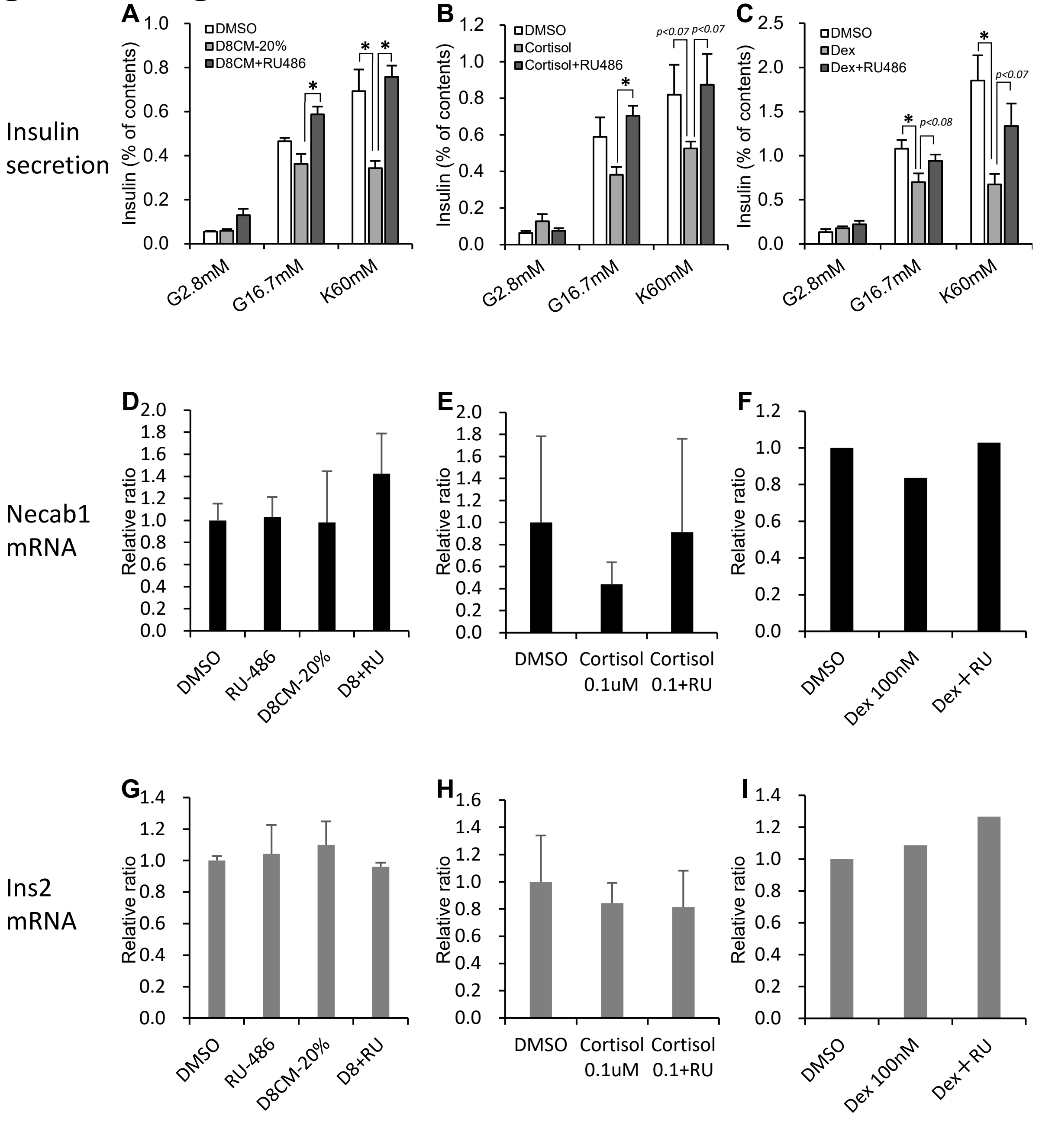

Fig. S6 Insulin secretion from islet of C57BL/6J mice exposed to D8CM

(A-C) Insulin secretion from isolated islets from mice fed a normal chow diet and exposed to D8CM- 20% (A), 0.5 μM cortisol (B), 10 μ M Dex (C), and/or 1 μ M RU-486, for 24 h were measured after treating with 2.8 mM glucose, 16.7 mM glucose, and 60 mM KCl. *: p<0.05. Values are shown as means±s.e.m. (n=3).

(D-F) Relative *Necab1* mRNA levels in isolated islets exposed to 20% D8CM (D) (n=3), 0.5 μ M cortisol (E) (n=3) and 100 μ M Dex (F) (n=1) and/or 1 μ M RU-486, for 24 h were detected by qPCR. *: p<0.05 vs NC. Values are shown as means±s.e.m. (n=4).

(G-I) Relative *Insulin2* mRNA levels in isolated islets exposed by 20% D8CM (G) (n=3), 0.5 μ M cortisol (H) (n=3), and 100 μ M Dex (I) (n=1) and/or 1 μ M RU-486, for 24 h were detected by qPCR. *: p<0.05 vs NC. Values are shown as means±s.e.m. (n=4).

Fig. S7 Udagawa

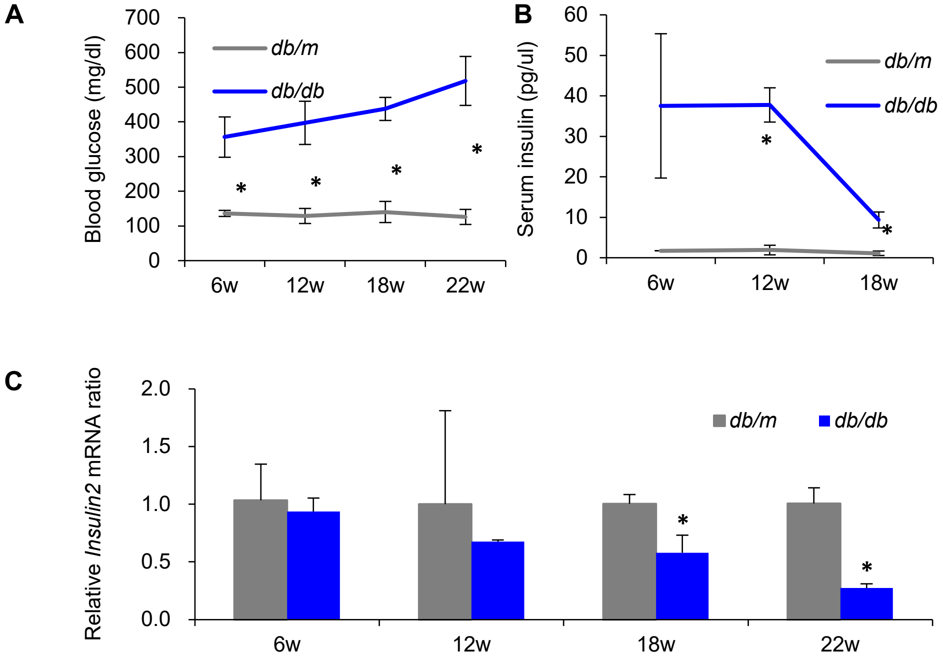

Fig. S7 Physiological analysis of *db/db* mice and control *db/m* mice
Fed blood glucose levels (A) and serum insulin (B) levels was examined in male *db/db* mice and *db/m* mice at 6, 12 and, 18 weeks-old (w). (C) The expression of *Insulin 2* mRNA levels in *db/m* and *db/db* mice at 6, 12, 18, 22 w was examined by qPCR. Values are shown as means±s.e.m. (n=4). *: $p<0.05$ vs *db/m* mice.

Fig. S8 Udagawa

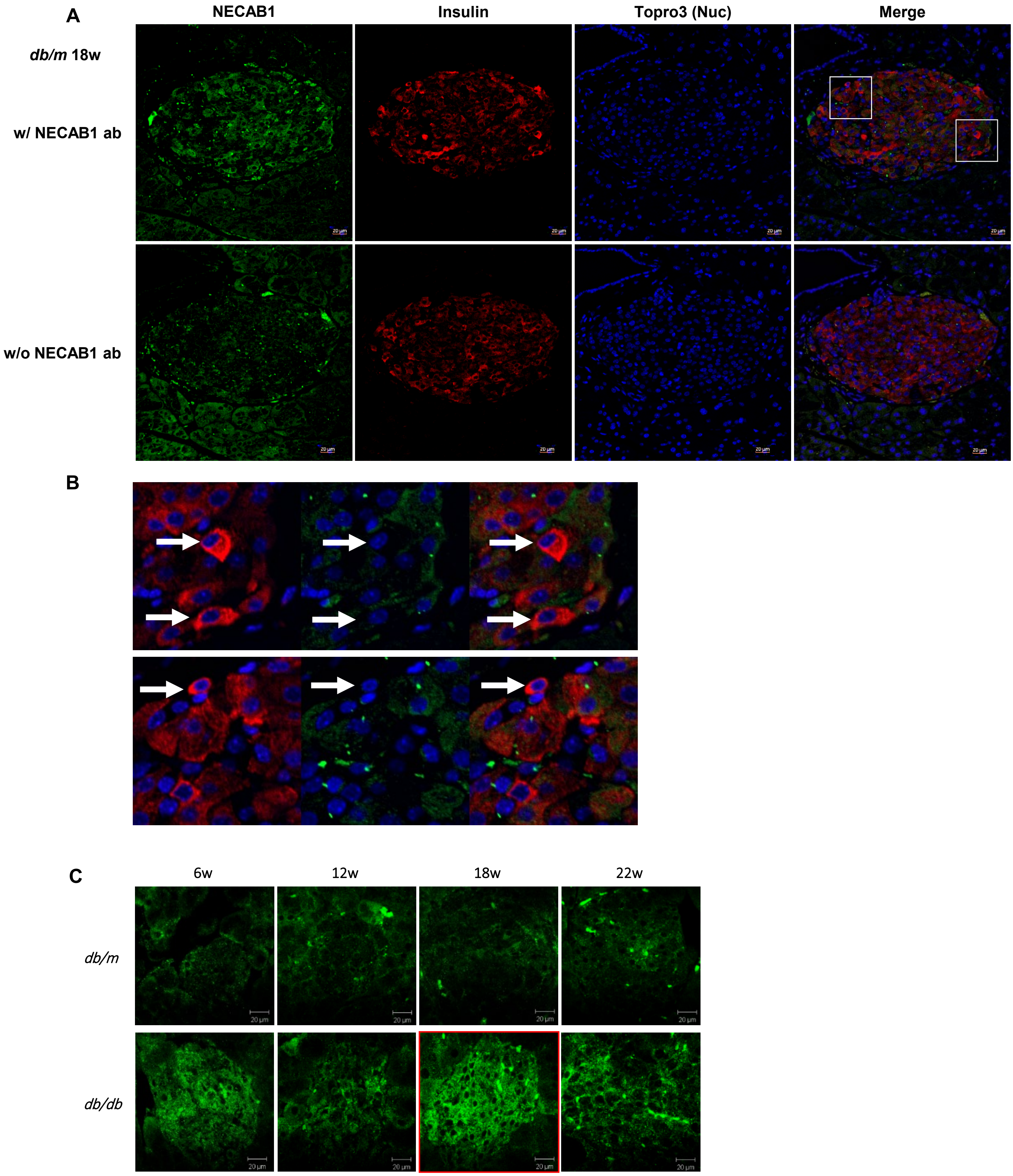

Fig. S8 Validation of NECAB1 antibody specificity.

(A) Fluorescence immunostaining was performed on sections of 18-week-old *db/db* mice with(w/) and without (w/o) NECAB1 antibody.

(B) The white squares show that β-cells with high level expression of insulin express NECAB1 at a lower level.

(C) The expression of NECAB1 was immunostained to detect GFP (green) using anti-NECAB1 antibodies in *db/db* mice and *db/m* mice at 6, 12, 18, 22 w. The immunostaining image of NECAB1 in *db/m* and *db/db* mice at 18 w is the same image as shown in Fig. 4C. Scale bar = 20 µm.

Fig. S9 Udagawa

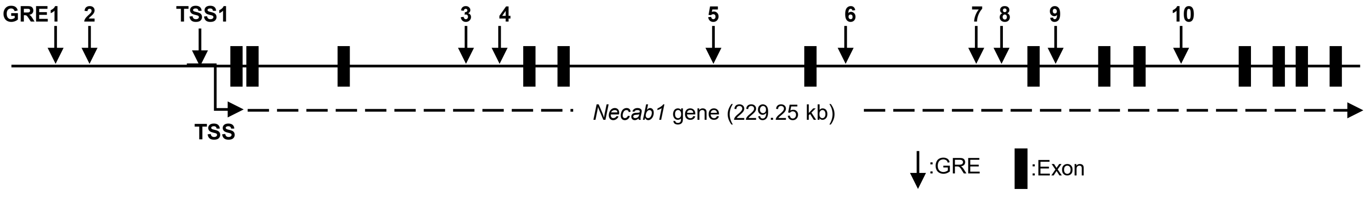

Fig. S9 *in silico* analysis of GRE in Necab1 enhancer region
Schema of the predicted area of GRE1-10 (arrows) 230 kbp upstream to 200 kbp downstream of the TSS in the rat Necab1 gene. See Table S4 for the details of the relevant genomic region.

Fig. S10 Udagawa

Fig. 2C

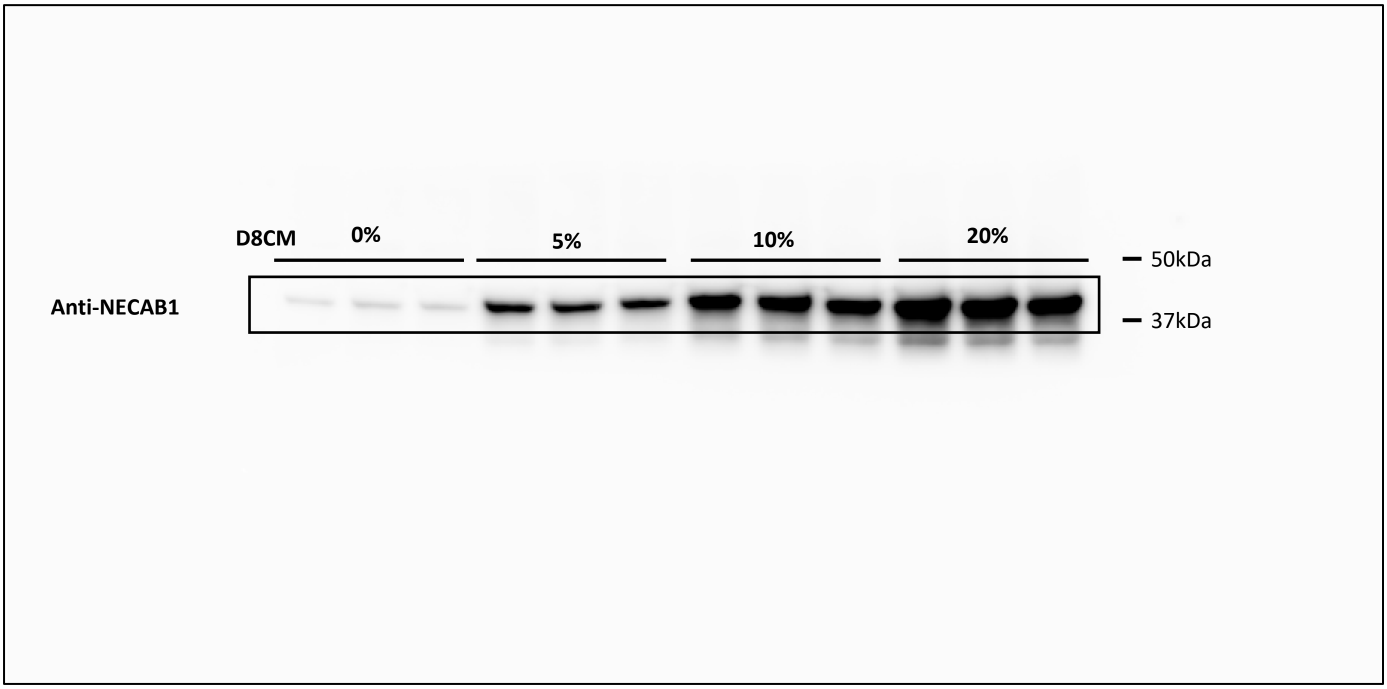

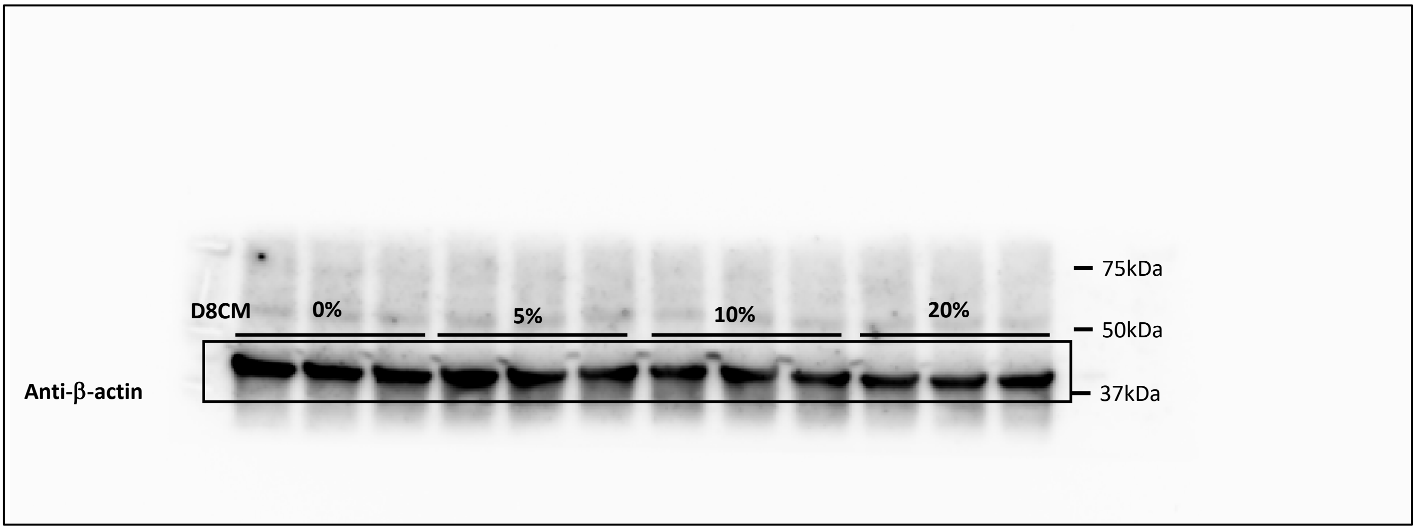

Fig. S10 Uncropped images of key panels in main figures. Black boxes indicate the cropped portion of each immunoblot presented in the corresponding main figure. Membranes were cut between 37 kda and 75 kda before hybridization with primary antibodies.

Fig. S11 Udagawa

Fig. 3F

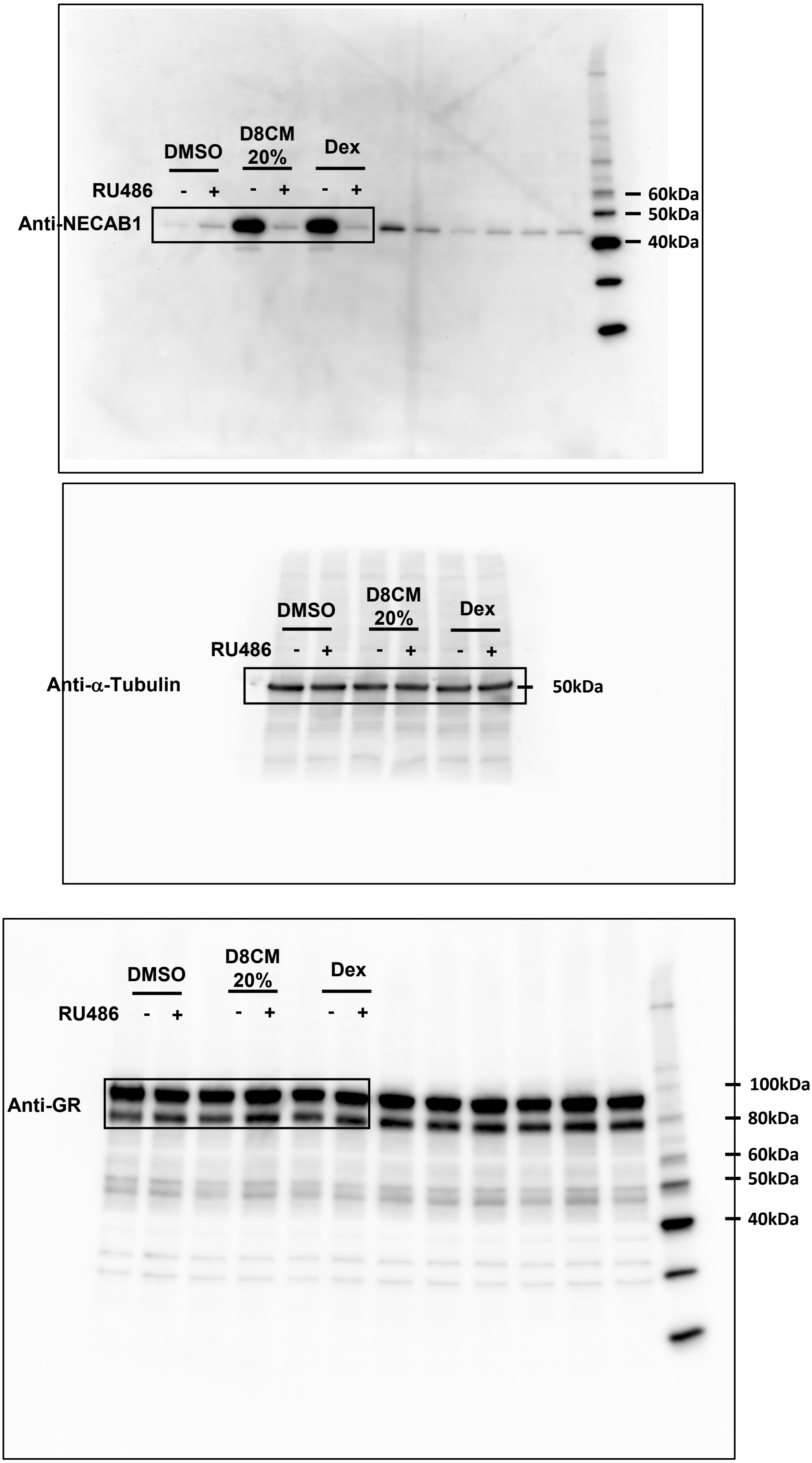

Fig. S11 Uncropped images of key panels in main figures. Black boxes indicate the cropped portion of each immunoblot presented in the corresponding main figure. Membranes for α-tubulin were cut between 20 kda and 100 kda before hybridization with primary antibodies.

Fig. S12 Udagawa

Fig. 4C

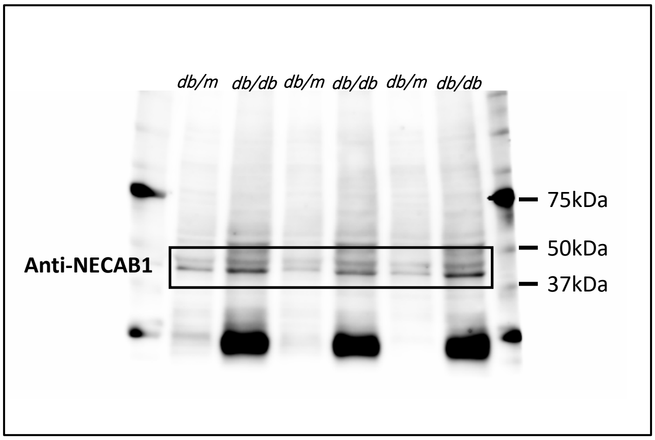

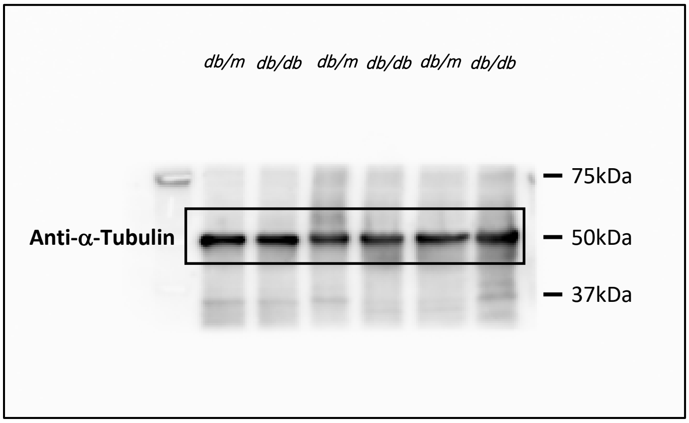

Fig. S12 Uncropped images of key panels in main figures. Black boxes indicate the cropped portion of each immunoblot presented in the corresponding main figure. Membranes for NECAB1 were cut between 15 kda and 250 kda before hybridization with primary antibodies. Membranes for α-tubulin were cut between 37 kda and 75 kda before hybridization with primary antibodies.

Fig. S13 Udagawa

Fig. S1C

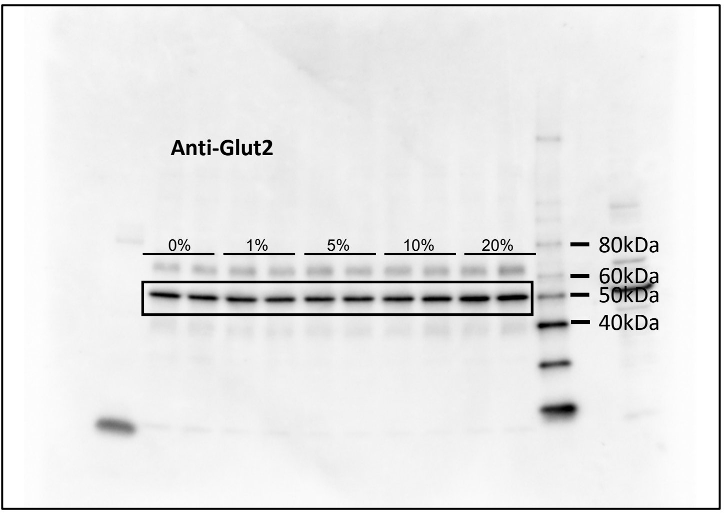

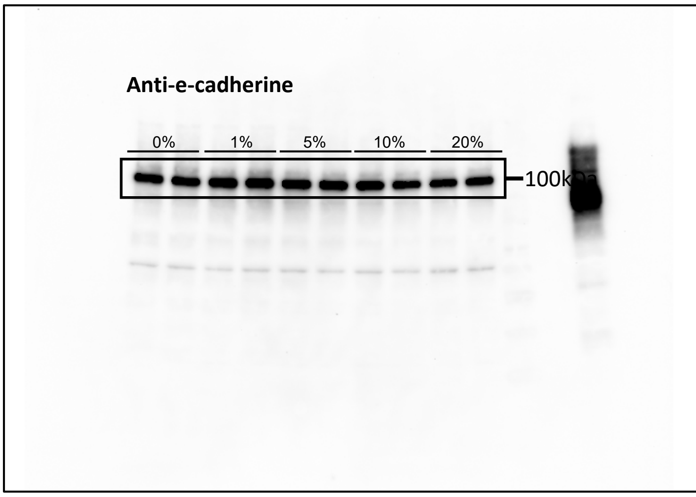

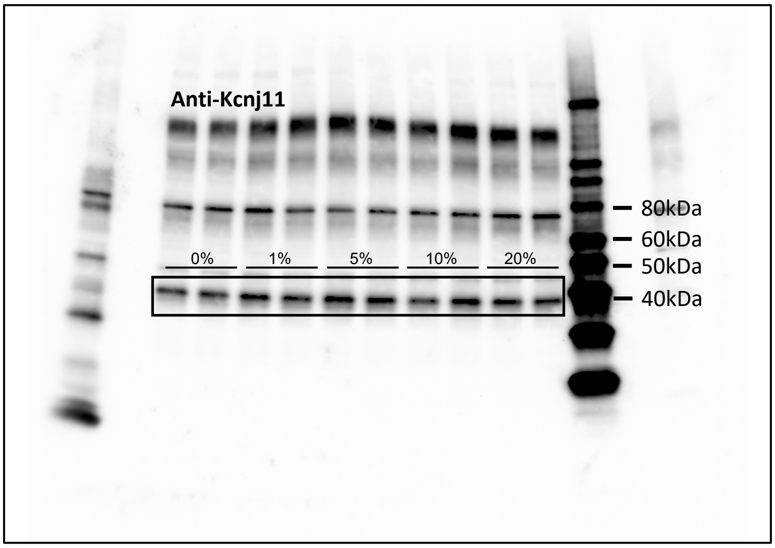

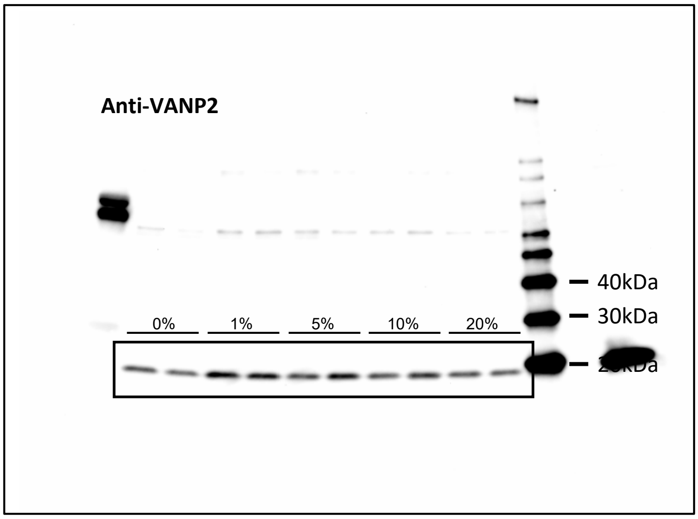

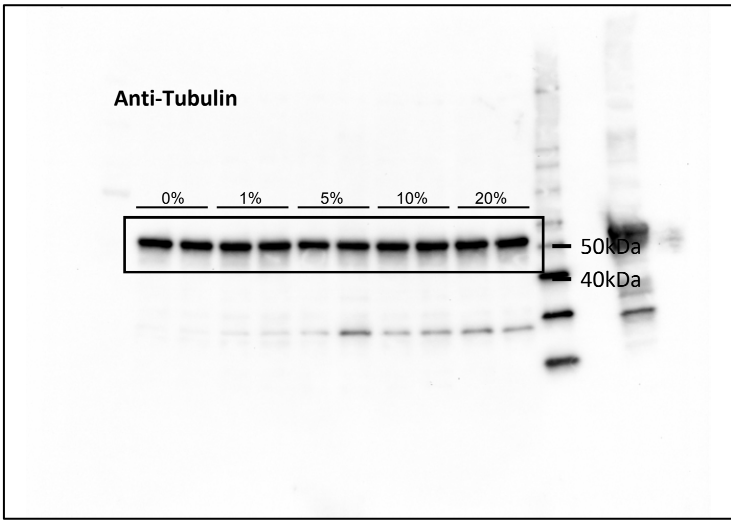

Fig. S13 Uncropped images of key panels in main figures. Black boxes indicate the cropped portion of each immunoblot presented in the corresponding main figure.

Fig. S14 Udagawa

Fig. S2C

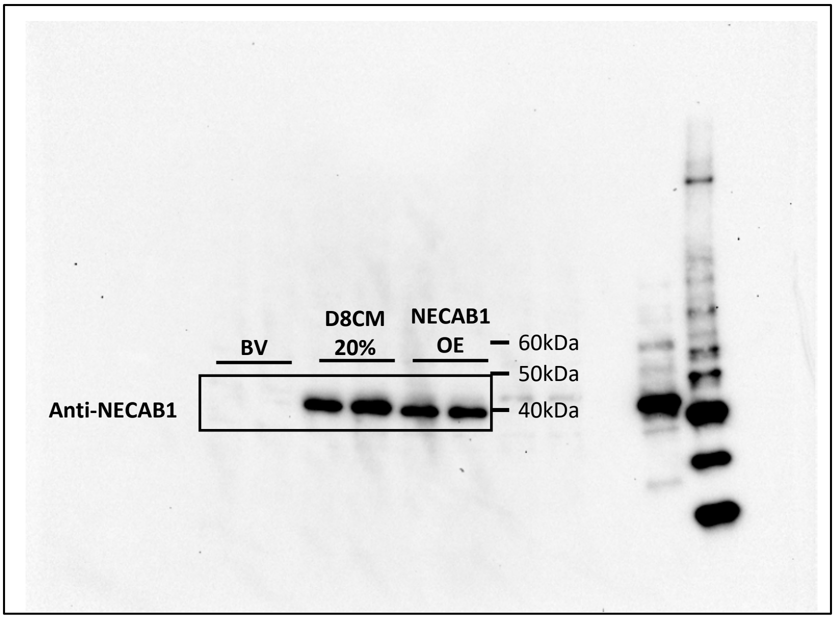

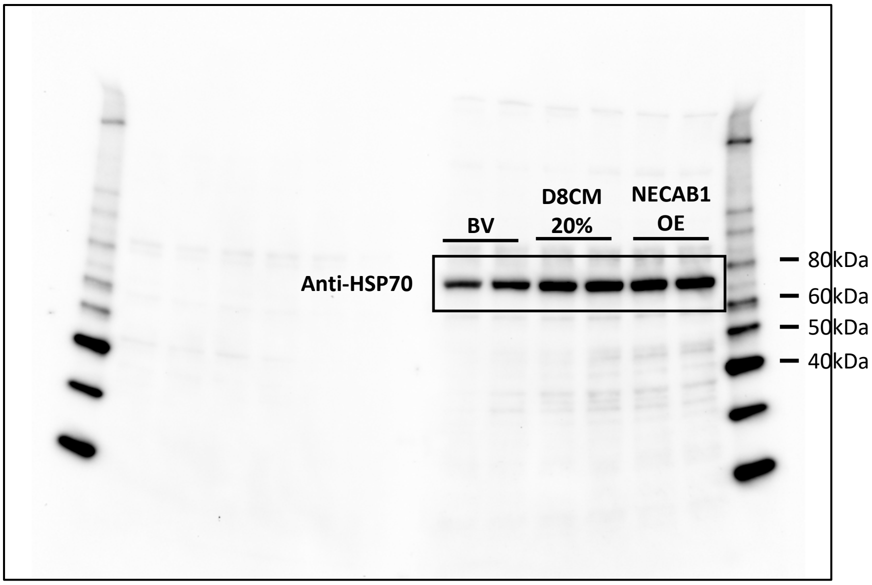

Fig. S14 Uncropped images of key panels in main figures. Black boxes indicate the cropped portion of each immunoblot presented in the corresponding main figure.

Fig. S15 Udagawa

Fig. S3A

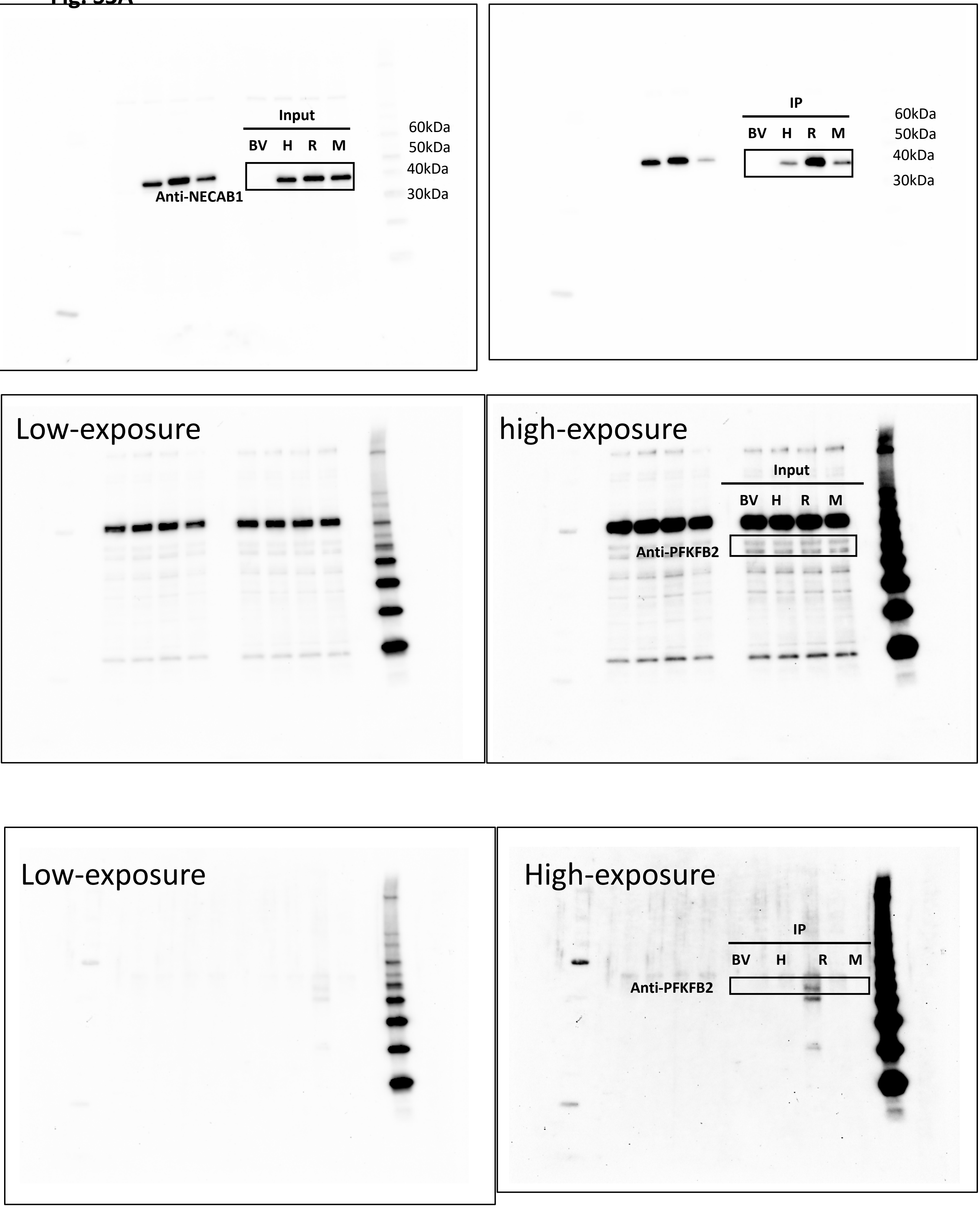

Fig. S15 Uncropped images of key panels in main figures. Black boxes indicate the cropped portion of each immunoblot presented in the corresponding main figure. Blot images in the Anti-PFKFB2 antibody were taken with high exposures to clarify the bands.

Fig. S16 Udagawa

Fig. S3C

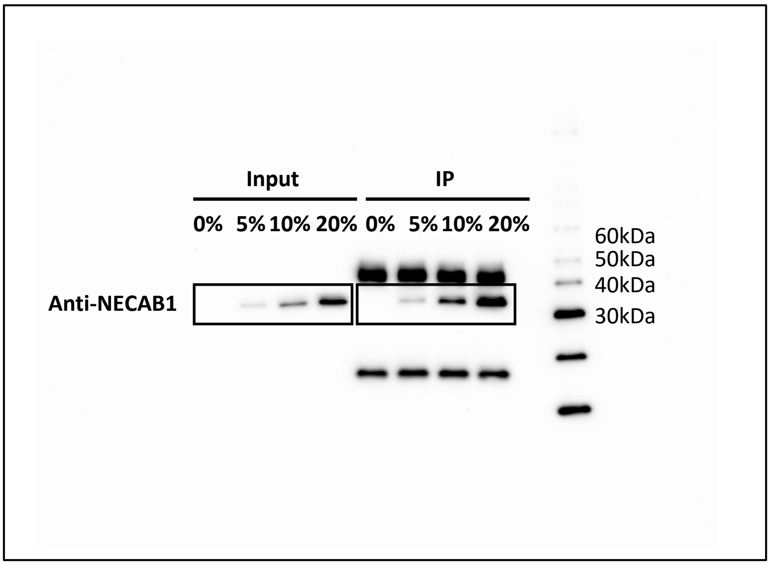

High-exposure

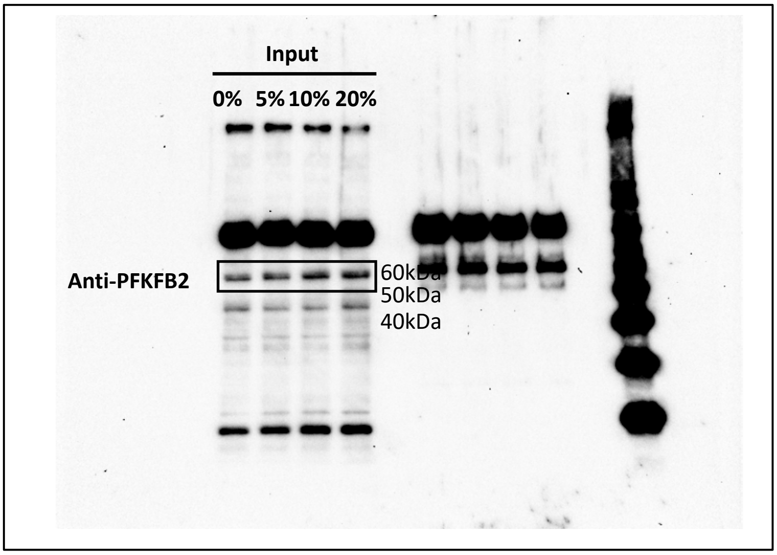

Low-exposure

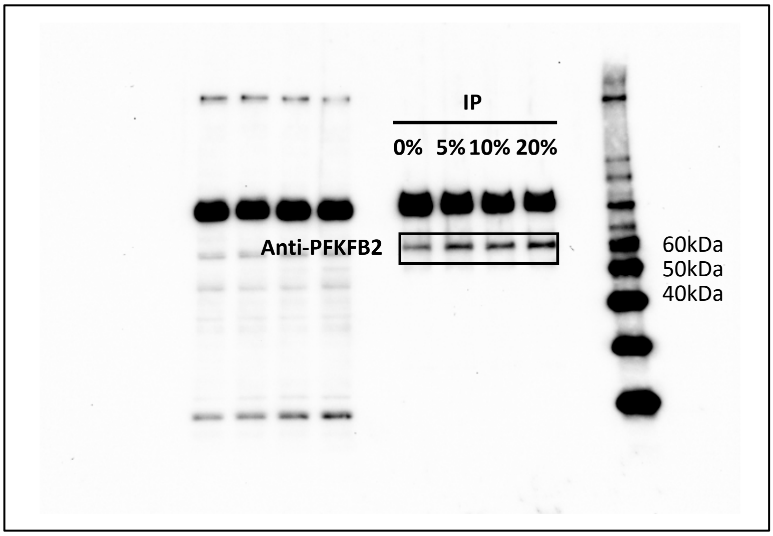

Fig. S16 Uncropped images of key panels in main figures. Black boxes indicate the cropped portion of each immunoblot presented in the corresponding main figure. Blot images of input sample in the Anti-PFKFB2 antibody were taken with high exposures to clarify the bands.

Table S1 MicroArray analysis increased data rank (Top 10)

Rank	Affymetrix Gene ID	NC1 vs D8CM1 Signal	NC1 vs D8CM1 Signal Log Ratio	NC1 vs D8CM1 Change p-value	NC2 vs D8-CM2 Signal	NC2 vs D8CM2 Signal Log Ratio	NC2 vs D8CM2 Change p-value	Gene Name to DAVID analysis
1	1386119_at	2207.6	6	0.00002	2248	6.1	0.00002	EST BE110033
2	1372213_at	2284.1	5.2	0.00002	2030.8	4.9	0.00002	similar to hypothetical protein MGC6835
3	1368401_at	715	4.8	0.00002	737.1	3.8	0.00002	glutamate receptor, ionotropic, AMPA 2
4	1369871_at	940.6	4.7	0.00002	906.6	4.6	0.00002	amphiregulin
5	1368144_at	1433.3	4	0.00002	1459.7	4.5	0.00002	regulator of G-protein signaling 2
**6**	**1369540_at**	**243.8**	**3.7**	**0.00002**	**243**	**3.3**	**0.00002**	**N-terminal EF-hand calcium binding protein 1**
7	1378156_at	200.6	3	0.000189	256.9	3.3	0.000147	similar to cystin 1
8	1387223_at	357.7	2.8	0.00002	355.6	2.9	0.00002	aminoadipate aminotransferase
9	1370606_at	1083.2	2.6	0.00002	1014.1	2.8	0.00002	purinergic receptor P2Y, G-protein coupled 1
10	1387154_at	7945	2.6	0.00002	7118.2	2.5	0.00002	neuropeptide Y

NC：Negative control, D8CM: Day8 conditioned medium from 3T3-L1

Table S2 MicroArray analysis decreased data rank (Top 10)

Rank	Affymetrix Gene ID	NC1 vs D8CM1 Signal	NC1 vs D8CM1 Signal Log Ratio	NC1 vs D8CM1 Change p-value	NC2 vs D8-CM2 Signal	NC2 vs D8CM2 Signal Log Ratio	NC2 vs D8CM2 Change p-value	Gene Name to DAVID analysis
1	1384679_at	4.5	-5.1	0.998664	22.3	-1.5	0.999135	similar to CG10806-PB, isoform B
2	1392382_at	4.7	-4.5	0.99998	4.4	-3.9	0.99998	transforming growth factor, beta 2
3	1387172_a_at	5.2	-4.2	0.999508	4.6	-4.5	0.999226	transforming growth factor, beta 2
4	1398431_at	65	-3.9	0.99998	59.6	-3.9	0.99998	carbonic anhydrase 8
5	1393841_at	0.5	-3.7	0.999693	3.3	-1.5	0.99996	similar to hypothetical protein FLJ31810
6	1398567_at	3.8	-3.3	0.999911	4.5	-3.7	0.999034	similar to FLJ42986 protein
7	1376425_at	12.2	-3.2	0.99998	5.7	-3.4	0.999973	transforming growth factor, beta 2
8	1381350_at	134.4	-3.1	0.99998	145.9	-3.1	0.99998	inhibitor of DNA binding 4
9	1371731_at	84.6	-3.1	0.99998	59.8	-2.9	0.99998	similar to Coatomer gamma-2 subunit (Gamma-2 coat protein) (Gamma-2 COP)
10	1375120_at	118.2	-2.9	0.999977	106.7	-2.8	0.99998	inhibitor of DNA binding 4

Table S3 Number of Unique Peptides and MASCOT Score by Proteomic Analysis

Sample No.		Protein	gene	MW	Score	Peptide	Coverage	Accession	*Note**
1	1	N-terminal EF-hand calcium-binding protein 1	Necab1 Efcbp1, Stip	40,904	109	4	9	Q9ESB5	*gi/11560137*
2	1	*Serum albumin [Bos taurus (Bovine)]*	ALB	69,293	708	18	35	P02769	*gi/1351907*
3	1	rCG64260, partial (Immunoglobulin)		12,786	101	2	17		*gi/149037302*
	2	immunoglobulin kappa-chain, partial		11,649	41	1	7		*gi/204795*
4	1	6-phosphofructo-2-kinase/fructose-2,6-bisphosphatase 2	Pfkfb2	54,823	60	6	11	Q9JJH5	*gi/77020248*

Note：Accession in Mascot Search Results*

Table S4 List of biological matters examined for the effects on the expression of *Necab1* in INS-1 cells

Stimulate	*Necab1* mRNA expression
Palmitate 500uM	No change
Chemically Defined Lipids concentrate (GIBCO #11905)	No change
Glucose 3mM	No change
Glucose 25mM	No change
Thapsigargin 50, 100nM	No change
Staurosporine 5, 50nM	No change
Amlodipine 5uM (Calcium channel inhibitor)	No change
BayK8644 10uM (Calcium channel activator)	No change
Forskolin	No change
Ca 10uM	No change
Ca 100uM	No change
ATP 100uM	No change
C2-ceramide	No change

Table S5 Putative GREs in the rat *Necab1* locus identified by JASPAR CORE database

GRE No.	Sequence	Score	Distance from TSS
pGRE-1	atgGGAACAGAGTGTTCTgtt	0.953	-42338
pGRE-2	ggaAGCACACAAAGTACTgag	0.955	-33441
pGRE-3	atgGGGACAGAGTGTTCTgct	0.951	62118
pGRE-4	agcAGAACACTCTGTCCCcat	0.966	72156
pGRE-5	agcAGAACACTCTGTCCCcat	0.966	120681
pGRE-6	atgAGCACAGAGTGTTCTgct	0.972	146798
pGRE-7	atgGGGACAGAGTGTTCTgct	0.951	173383
pGRE-8	atgGGGACAGAGTGTTCTgct	0.951	174708
pGRE-9	agcAGAACACTCTGTCCCcat	0.951	183459
pGRE-10	atgGGGACAGAGTGTTCTgct	0.951	208819
consensus GRE	AGAACANNNTGTTCT		
TSS location	Chr. 5; 29427273		

Score: JASPAR CORE database

Table S6 Primers used for cloning for rat NECAB1 promoter

Cloning for rat NECAB1 promoter		Sequence	Products size
ratNECAB1 (-5000+70) Fw	forward	5'-GGGGTACCAGCCTGTACTC AGTAATTCATTCATTACTCTC-3'	5088 bp
ratNECAB1 (-5000+70) Rv	reverse	5'-CCTAGCTAGCCCTCAGCAAC CCTGGCGCCTGGCGGGCAGAGA-3'	

Table S7 Primers used for cloning of rat GRE in *Necab1* promoter

Primer name	Strand	Sequence
GRE1-oligo	forward	5'-CatgGGAACAGAGTGTTCTgttC-3'
	reverse	5'-TCGAGaacAGAACACTCTGTTCCcatGGTAC-3'
GRE2-oligo	forward	5'-CggaAGCACACAAAGTACTgagC-3'
	reverse	5'-TCGAGctcAGTACTTTGTGTGCTtccGGTAC-3'
GRE3-oligo	forward	5'-CatgGGGACAGAGTGTTCTgctC-3'
	reverse	5'-TCGAGagcAGAACACTCTGTCCCcatGGTAC-3'
GRE4-oligo	forward	5'-CagcAGAACACTCTGTCCCcatC-3'
	reverse	5'-TCGAGatgGGGACAGAGTGTTCTgctGGTAC-3'
GRE6-oligo	forward	5'-CatgAGCACAGAGTGTTCTgctC-3'
	reverse	5'-TCGAGagcAGAACACTCTGTGCTcatGGTAC-3'

Table S8 Primers used for cloning for rat Glucocorticoid receptor (GR) gene

Primer name	Strand	Sequence	Product size
GR	forward	5'-GCCGCGATCGCCATGGACTCC AAAGAATCCTTAGCTC-3'	2403 bp
	reverse	5'-CGTACGCGTTTTTTGATGAAAC AGAAGCTTTTTG-3'	

Table S9 Primers used for FAIRE-qPCR and ChIP-qPCR

Primer name	Strand	Sequence	Product size
GRE1-FAIRE	forward	5'-GTTTGCCAGGATTGCTGTAGA-3'	112 bp
	reverse	5'-CACAGCTGAAGACCAGTAGACAG-3'	
GRE1-ChIP	forward	5'-GATTGCTGTAGACCTGTTTTCCTT-3'	74 bp
	reverse	5'-CTACATGCCAAAAACAGAACACTC-3'	
GRE2-FAIRE	forward	5'-GTTGAACCAAGGAGAAGTGGCAG-3'	121 bp
	reverse	5'-GGAGAATCTCTTCCGGGCATCTG-3'	
GRE3-FAIRE	forward	5'-CTGACAGTGGCTAGACTGTCCT-3'	146 bp
	reverse	5'-AGAGGAAAAACTACACGCCGGA-3'	
GRE4-FAIRE	forward	5'-CTCGGAATCACAGGTAAGACCAAC-3'	179 bp
	reverse	5'-GAGTGCTGTAGACCTGTTTTCCTG-3'	
GRE5-FAIRE	forward	5'-ACCTGTAGAGAGGAAAAACTACATGC-3'	136 bp
	reverse	5'-CTGCCATTTCTGACAGTGGCTAG-3'	
GRE6-FAIRE	forward	5'-CTATAGGCCTGTGTGTCAGGAGTG-3'	160 bp
	reverse	5'-CAACCTGCCTGGTGGACTCAG-3'	
GRE7-FAIRE	forward	5'-GATGGGAGAATTGGGCTCTGATG-3'	254 bp
	reverse	5'-GAACAGCTGAAAACCTGTAGAGAG-3'	
GRE8-FAIRE	forward	5'-GTTCTCCATGTCTTTCTTGAAGTCCTC-3'	432 bp
	reverse	5'-GCCTCAGGACCACAGGTAAGA-3'	
GRE9-FAIRE	forward	5'-CACGAGCAAACCTGAGCCTC-3'	330 bp
	reverse	5'-CTCCGATGATGGCATGTAGTCTTG-3'	
GRE10-FAIRE	forward	5'-GTGTGTTCATGTCTTTCTTGAAGTCCT-3'	341 bp
	reverse	5'-GTAGAGAGGAAAAACTACACGCCTG-3'	
TSS-FAIRE and ChIP	forward	5'-ACTCAGATTCCTTCTTAAGGCTCA-3'	90 bp
	reverse	5'-GATAGACCAGATGGAGCTTTGTTT-3'	
Nanog-FAIRE	forward	5'-TCCTCTGGGGACCTACCTCT-3'	143 bp
	reverse	5'-ACAGTCCGCATCTTCTGCTT-3'	
